# Unraveling the Sex Chromosome Heteromorphism of the Paradoxical Frog *Pseudis tocantins*

**DOI:** 10.1371/journal.pone.0156176

**Published:** 2016-05-23

**Authors:** Kaleb Pretto Gatto, Carmen Silvia Busin, Luciana Bolsoni Lourenço

**Affiliations:** 1 Departamento de Biologia Estrutural e Funcional, Instituto de Biologia, Universidade Estadual de Campinas, 13083–863, Campinas, São Paulo, Brasil; 2 Laboratório de Citogenética, Instituto de Ciências Biológicas, Universidade de Passo Fundo, 99001–970, Passo Fundo, Rio Grande do Sul, Brasil; Universita degli Studi di Roma La Sapienza, ITALY

## Abstract

The paradoxical frog *Pseudis tocantins* is the only species in the Hylidae family with known heteromorphic Z and W sex chromosomes. The Z chromosome is metacentric and presents an interstitial nucleolar organizer region (NOR) on the long arm that is adjacent to a pericentromeric heterochromatic band. In contrast, the submetacentric W chromosome carries a pericentromeric NOR on the long arm, which is adjacent to a clearly evident heterochromatic band that is larger than the band found on the Z chromosome and justify the size difference observed between these chromosomes. Here, we provide evidence that the non-centromeric heterochromatic bands in Zq and Wq differ not only in size and location but also in composition, based on comparative genomic hybridization (CGH) and an analysis of the anuran PcP190 satellite DNA. The finding of PcP190 sequences in *P*. *tocantins* extends the presence of this satellite DNA, which was previously detected among Leptodactylidae and Hylodidae, suggesting that this family of repetitive DNA is even older than it was formerly considered. Seven groups of PcP190 sequences were recognized in the genome of *P*. *tocantins*. PcP190 probes mapped to the heterochromatic band in Wq, and a Southern blot analysis indicated the accumulation of PcP190 in the female genome of *P*. *tocantins*, which suggests the involvement of this satellite DNA in the evolution of the sex chromosomes of this species.

## Introduction

In vertebrates, sex determination may be triggered by genetic factors (genetic sex determination, GSD) or environmental factors (environment sex determination, ESD) (reviewed in [1] and [2]). Sex chromosomes are present in organisms with GSD, resulting in male heterogamety (as observed in mammals) or female heterogamety (common in birds and snakes). In amphibians, both male and female heterogamety are present (reviewed in [3] and [4]), but heteromorphic sex chromosomes may be recognized in only a few species [4, 5]. Among anurans, approximately 40 species display heteromorphic sex chromosomes, which include cases of multiple sex chromosome systems (e.g., [6]) and 00/0W system [7], besides the usual XX/XY and ZZ/ZW systems (reviewed in [4]). Furthermore, the origin and differentiation of sex chromosomes in the order Anura are recurrent phenomena, with female heterogamety being the inferred ancestral condition in the order [8]. Consequently, the anurans are an interesting group for studying the evolution of sex chromosomes.

In most cases found in Anura, sex chromosomes may be recognized only after the use of banding techniques (e.g., [4, 9, 10]). Few anuran karyotypes have been analyzed by CGH [11–13] despite the utility of this technique in important advances in the research on sex chromosomes in other groups (e.g., [14–16]).

The accumulation/amplification of satellite DNA (sat DNA) in heterochromatin segments has been related to the differentiation of sex chromosomes in many taxa of animals and plants, probably due to a putative role of this class of repetitive DNA in suppressing recombination [17–22]. For anuran species, only a few satellite DNA sequences have been isolated, characterized and mapped cytogenetically (e.g., [23–25]) and PcP190 is one of them [26, 27]. The PcP190 family of satellite (sat) DNA is likely derived from 5S ribosomal DNA (5S rDNA) and was first isolated from the genome of the leptodactylid frog *Physalaemus cuvieri* [27]. This sat DNA may be largely distributed among anurans because PcP190 sequences were already found in other species of *Physalaemus*, in the leptodactylid genus *Leptodactylus* and also in the genus *Crossodactylus*, a representative of the Hylodidae family [26]. The amount of PcP190 sequences varied among the genomes studied and, in karyotypes of *Physalaemus*, this sat DNA was mapped by *in situ* hybridization to heterochromatic regions, including an interstitial band that was differentially detected in the Z and W chromosomes of *Physalaemus ephippifer* [26]. Accordingly, the mapping and characterization of PcP190 sequences constitute a promising approach to anuran cytogenetics.

The hylid genus *Pseudis* is an interesting group for the study of sex chromosome evolution. Among the seven species of this genus, only *Pseudis tocantins* Caramaschi and Cruz [28] has heteromorphic Z and W sex chromosomes [29]. The Z chromosome of *P*. *tocantins* is metacentric and bears an interstitial nucleolar organizer region (NOR) on the long arm, which is adjacent to a pericentromeric heterochromatic band. In contrast, the submetacentric W chromosome of this species carries a pericentromeric NOR on the long arm, adjacent to a clearly evident heterochromatic band. This band is greater than the band found on the Z chromosome and justify to the size difference between these sex chromosomes. The distinct relative position of the NOR and the heterochromatin band in the long arm of the Z and W chromosomes of *P*. *tocantins* suggest the occurrence of an inversion event during the evolution of these chromosomes [29]. However, little is known about the heterochromatic blocks that differ in size between these Z and W chromosomes, although their distinct sizes suggest the occurrence of amplification or accumulation events of repetitive DNA.

To better evaluate the differences between the Z and W chromosomes of *Pseudis tocantins*, we used comparative genomic hybridization (CGH) and isolated, characterized and cytogenetically mapped sequences belonging to the PcP190 sat DNA family and 5S rDNA.

## Materials and Methods

### Individuals and chromosome preparations

We used the chromosome preparations and tissue samples that were previously obtained by Busin et al. [29] from eleven individuals of *Pseudis tocantins* (four males and seven females), collected from the Porto Nacional, state of Tocantins, Brazil. The specimens are deposited in the Natural History Museum of Zoology “Prof. Adão José Cardoso”, at the University of Campinas (ZUEC), under the accession numbers 13227–13234, or in the National Museum of Rio de Janeiro (MNRJ), under the accession numbers 35456–35458.

### Comparative genomic hybridization

Genomic DNA was extracted from liver samples of female and male specimens of *Pseudis tocantins* according to Medeiros et al. [30]. DNA integrity was analyzed by electrophoresis in a 0.8% agarose gel and quantified using a Nanodrop spectrophotometer (Thermo Scientific). To obtain genomic probes, female and male genomic DNA samples (1 μg) were labeled with Cy3-dCTP (GE Healthcare) and FITC-12-dUTP (Roche), respectively, using a Nick Translation Kit (Roche). The two probes were precipitate with 4 μg of boiled competitor DNA obtained from genomic male DNA. To obtain the competitor DNA, male genomic DNA in 0.3 M-NaCl was boiled in an autoclave for 30 minutes at 1.4 atm/120°C, resulting in fragments of 75–500 bp. Then, the DNA fragments were frozen in liquid nitrogen, treated with phenol:chloroform, precipitated with 2.5 volumes of 100% ethanol and resuspended in Milli-Q water.

### PcP190 and 5S rDNA isolation, cloning and sequencing

Sequences belonging to the PcP190 satellite DNA family were isolated by PCR from female and male genomic DNA and from microdissected Z and W chromosomes of *Pseudis tocantins* using the primers P190F (5’-AGACTGGCTGGGAATCCCAG-3’) and P190R (5’-AGCTGCTGCGATCTGACAAGG-3’) as described by Vittorazzi et al. [27].

For the microdissection of the chromosomes we dropped cell suspensions onto slides covered with a polyethylenenaphtalene (PEN) membrane previously that was exposed to UV light to avoid contamination. Chromosome preparations were stained with 10% Giemsa and microdissection was performed using a PALM laser system (Zeiss) equipped with an oil immersion 100x objective. The laser intensity used to cut the membrane was 0.5–0.6 μJ/pulse, and each isolated islet was catapulted to the lid of a microtube (0.2 mL) containing 1 μL of mineral oil and using a single pulse of 0.2 μJ. The collected material was used in PCR with the primers P190F and P190R using Illustra PuReTaq Ready-To-Go (GE Healthcare). In some experiments, the microdissected material was first amplified using GenomePlex Single Cell WGA4 (Sigma-Aldrich), and the resulting products were subsequently submitted to PCR with the primers P190F and P190R.

Because the PcP190 satellite DNA is derived from 5S rDNA [27], we analyzed 5S rDNA sequences from *Pseudis tocantins*, which were isolated by PCR with the primers 5S-A (5’-TACGCCCGATCTCGTCCGATC-3’) and 5S-B (5’–CAGGCTGGTATGGCCGTAAGC–3’) [31]. The amplified fragments of the PcP190 and 5S rDNA sequences were analyzed by electrophoresis in 1% agarose gel, purified using the Wizard SV Gel and PCR Clean-up System (Promega), ligated into pGEM-T Easy Vector (Promega) and introduced into an *E*. *coli* JM109 strain employing the TransformationAid Bacterial Transformation Kit (Fermentas), following the manufacturer’s instructions. Recombinant colonies were identified and plasmid extraction was performed using the mini-prep method described by Sambrook and Russel [32].

Cloned fragments were amplified by PCR with the universal primers T7 and SP6, purified using the Wizard SV Gel and PCR Clean-up System (Promega) and sequenced using the BigDye Terminator Kit (Applied Biosystems) following the manufacturer’s instructions. The reaction products were precipitated using 80% ethanol, centrifuged and then washed in 70% ethanol. The products were resuspended in loading dye, denatured and then sequenced on an automated sequencer (ABI PRISM® 3100 Genetic Analyzer-Hitachi), using the DNA sequencing facility of the Chemistry Institute at the University of São Paulo.

### Nucleotide sequence analyses

The PcP190 and 5S rDNA nucleotide sequences were edited using BioEdit 7.0.9.0 [33] and compared with each other, and with sequences from GenBank (www.ncbi.nlm.nih.gov). We estimated the similarity between sequences based on p- distance values that were calculated in MEGA 6 [34], except in the comparisons of the hypervariable regions of the PcP sequences, whose similarity values were inferred using Bioedit. Maximum likelihood analysis were performed using MEGA 6 [34] under the Kimura-2-parameter model with gamma distribution. Only the complete conserved region of the PcP190 sequences were used in the maximum likelihood analysis. Median joining network [35] was calculated using Network 4.6.1.3 (Fluxus Engineering). Haplotype data file for network analysis was generated in DnaSP 5.10 [36] not considering indels and invariable sites.

### Fluorescence *in situ* hybridization (FISH) of PcP190 and 5S rDNA probes

Fragments of PcP190 and 5S rDNA sequences obtained from *Pseudis tocantins* as described above were labeled with digoxigenin-12-dUTP (Roche) using the PCR Dig Probe Synthesis Kit (Roche). Labeled DNA was co-precipitated with sonicated salmon sperm DNA (100 ng/μL) using 3M sodium acetate (1/10 volume) and ethanol. The pellet was washed in 70% ethanol and resuspended in hybridization buffer (50% formamide, 2x SSC and 10% dextran sulfate). The hybridization protocol was performed according to Viegas-Péquignot [37]. Digoxigenin-labeled probes were detected using an anti-digoxigenin anti-body conjugated with rhodamine (Roche), following the manufacturer´s instructions. Chromosomes were stained with DAPI (0.5 μg/mL). For the analysis of the PcP-1a probes, a control experiment was done, which consisted in their hybridization with metaphase chromosomes of an exemplar of *Physalaemus* aff. *cuvieri* (ZUEC 17897). Images were captured on an Olympus Bx60 fluorescence microscope and edited using Adobe Photoshop CS3 or/and Image ProPlus 4.0 (Media Cybernetics).

### Detection of PcP sequences by Southern blotting

To estimate the abundance in male and female genome of the PcP190 sequences mapped by FISH exclusively to the W chromosome of *Pseudis tocantins* (PcP-1b and PcP-2; see Results for details) we used Southern blotting. Genomic DNA of *P*. *tocantins* males and females were separately digested with Ban I (recognition site: GGyrCC) and *Mbo*II (recognition site: GAAGA[N]8) endonucleases (Promega), whose restriction sites were found in the PcP-1b and PcP-190-2 sequence, respectively. Complete and partial digestions of the DNA samples were achieved using 16 or 4 hours for the endonuclease reaction. Restriction fragments were electrophoresed in a 1.2% agarose gel and transferred to a nitrocellulose membrane according to Sambrook and Russel [32]. Probes generated from the PcP-1b and PcP-2 sequences were obtained using the PCR Dig Probe Synthesis Kit (Roche) and hybridized overnight at 60°C to the restriction fragments on the nitrocellulose membrane. After hybridization, the nitrocellulose membranes were washed twice in 2x SSC/0.1% SDS (5 minutes in each wash) at 37°C and then washed twice in 0.1x SSC/0.1% SDS (15 minutes in each wash) at 37°C. Probes were detected using the DIG Nucleic Acid Detection Kit (Roche), following the manufacturer´s instructions.

## Results

### CGH

**Fig 1:**
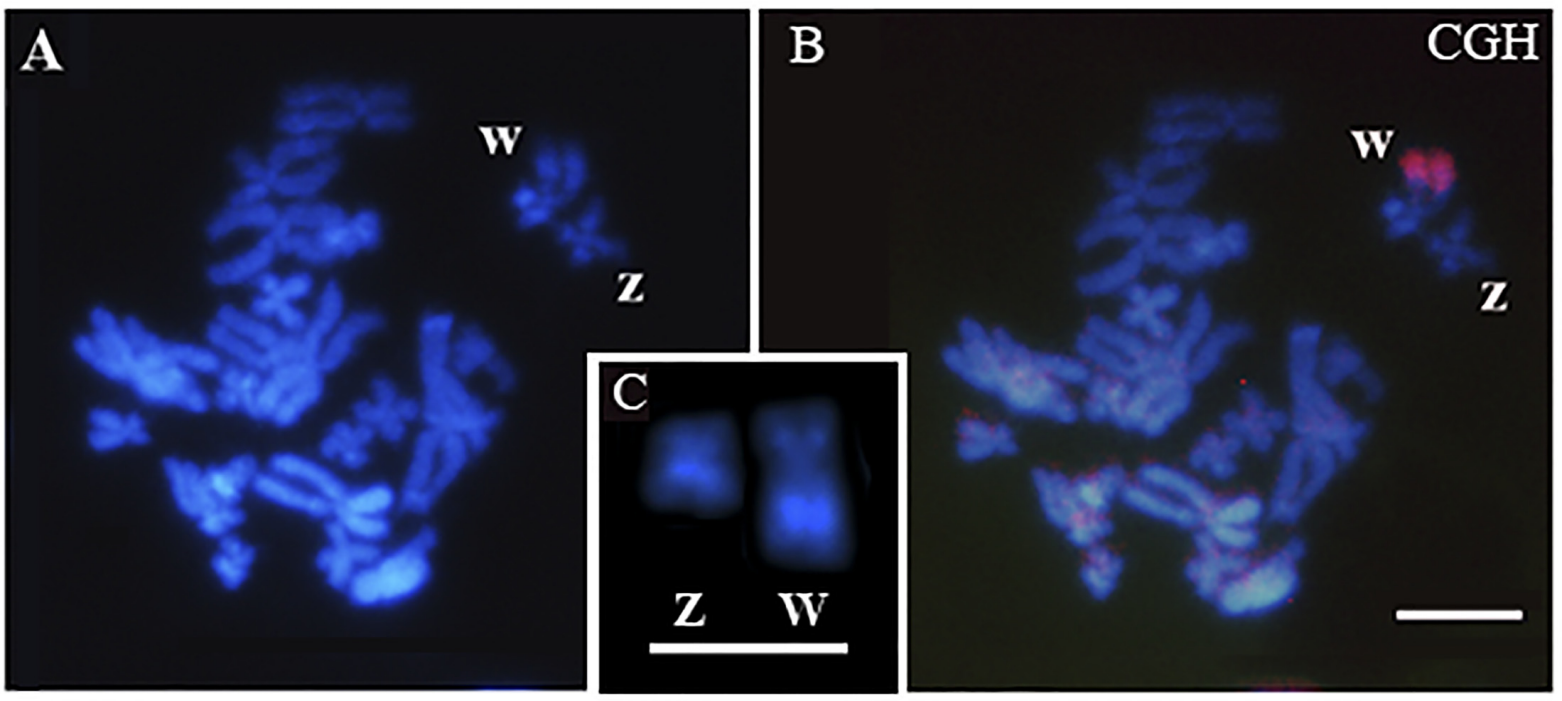
Comparative genomic hybridization on a female karyotype of *Pseudis tocantins*. (A-B) Using female genomic probe labeled with Cy-3 (red), male derived probe labeled with FITC (green), male unlabeled competitor DNA and DAPI counterstaining, CGH detected a Cy-3 signal in the W chromosome. In (A), DAPI-stained chromosomes are shown. (C**)** ZW pair after C-banding and DAPI staining. Note the heterochromatic bands in the Z and W chromosomes. Bar: 5 μm.

In the CGH experiments, a strong female-specific hybridization signal was observed at the heterochromatic block in the long arm of the W chromosome of *Pseudis tocantins* (Fig 1). No signal was observed on the Z chromosome or on the autosomes.

### 5S rDNA of *Pseudis tocantins*

**Fig 2:**
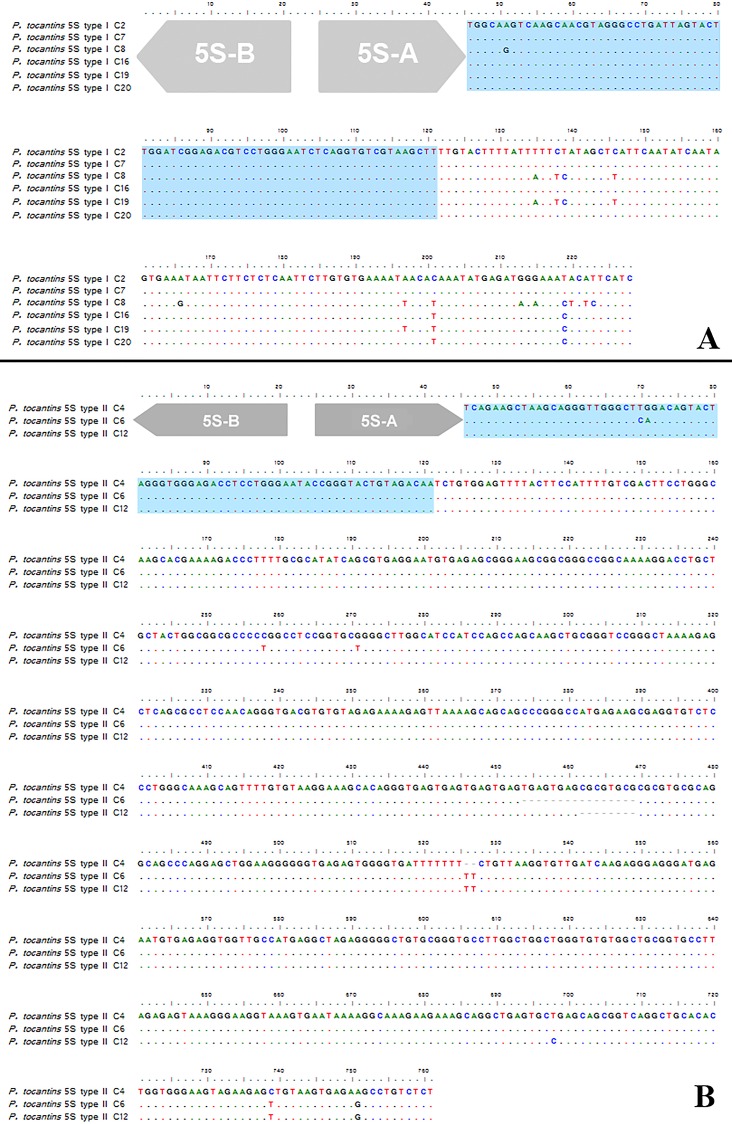
**Nucleotide sequence of the type I (A) and type II (B) 5S rDNA of *Pseudis tocantins*.** The shaded areas indicate the presumed transcribing box. The regions corresponding to the primers 5S-A and 5S-B used to obtain the *P*. *tocantins* sequences are indicated by arrows.

Among the nine cloned fragments of 5S rDNA, two types of sequence were recognized, which differed mainly in size and in composition of the presumed non- transcribed spacer (NTS). Type I 5S rDNA sequences had 107 bp in the presumed NTS region, while the NTS recognized in the type II 5S rDNA sequences had 625–639 bp (Fig 2). The six type I 5S rDNA sequences isolated from *Pseudis tocantins* were 97% similar, and the few differences among them were found mainly in the NTS region (Fig 2). Likewise, the type II 5S rDNA samples were highly similar to each other (99%, if the indels–see Fig 1 –are not considered; 97% if the indels are considered).

**Fig 3:**
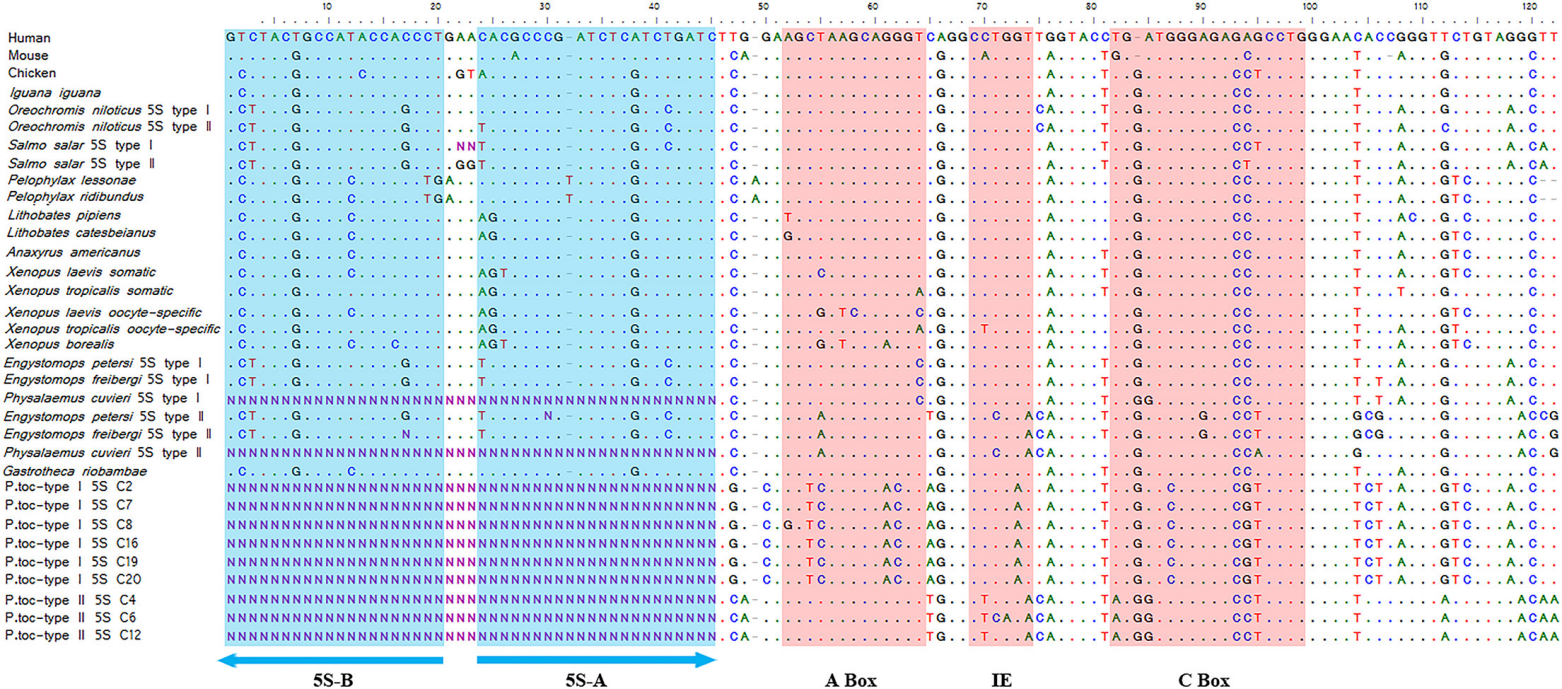
Presumed transcribing region of 5S rDNA. Presumed transcribing region of the repetitive units of type I (*Pseudis tocantins* type I-C2, *P*. *tocantins* typeI-C8) and type II 5S rDNA of *Pseudis tocantins* aligned with those from several anurans and other vertebrate species obtained from GenBank (Human 5S rRNA gene—K02217.1, Mouse 5S rRNA gene—K02235.1, Chicken 5S rRNA gene—X01309.1, *Iguana* 5S rRNA gene—M10817.1, *Oreochromis niloticus* type I 5S rRNA gene—AF478461.1, *O*. *niloticus* type II 5S rRNA gene—AF478462.1, *Salmo salar* type I 5S rRNA gene—S73107.1, *S*. *salar* type II 5S rRNA gene—S73106.1, *Pelophylax lessonae* 5S rRNA gene—FJ572051.1, *Pelophylax ridibundus* 5S rRNA gene—FJ572052.1, *Lithobates pipiens* 5S rRNA gene—X58368.1, *Lithobates catesbeianus* 5S rRNA gene—X58367.1, *Anaxyrus americanus* 5S rRNA gene—X58365.1 (as *Bufo americanus*), *Xenopus laevis* somatic 5S rRNA gene—J01009.1, *X*. *laevis* oocyte specific 5S rRNA gene—J01010.1, *Xenopus tropicalis* somatic 5S rRNA gene—X12622.1, *X*. *tropicalis* oocyte specific 5S rRNA gene—X12623.1, *Xenopus borealis* 5S rRNA gene—V01425.1, *Engystomops petersi* type I 5S rRNA gene—JF325862.1, *Engystomops freibergi* type I 5S rRNA gene—JF325870.1, *Physalaemus cuvieri* type I 5S rRNA gene—JF281131.2, *E*. *petersi* type II 5S rRNA gene—JF325847.1, *E*. *freibergi* type II 5S rRNA gene—JF325845.1, *P*. *cuvieri* type II 5S rRNA gene—JF281131.2 and *Gastrotheca riobambae* 5S rRNA gene—M74438.1). Blue shaded areas correspond to the annealing sites of the primers 5SA and 5SB, used here to isolate the *P*. *tocantins* sequences and red shaded areas correspond to internal control region of the 5S rRNA gene.

Intriguingly, the presumed transcribed regions of the type I and type II 5S rDNA of *Pseudis tocantins* were not very similar in nucleotide composition (average similarity value = ~ 65%) (Fig 3, Table 1). When compared with the corresponding region of the 5S rDNA sequences of other anurans, the presumed transcribed region of the type I and type II 5S rDNA sequences of *P*. *tocantins* showed an average similarity of ~ 77% and ~79%, respectively. The presumed transcribed region of the type II 5S rDNA of *P*. *tocantins* is more similar to the type II 5S rDNA from *Physalaemus cuvieri*, while the type I 5S rDNA is more similar to the 5S rRNA gene from *Anaxyrus americanus* (Table 1).

**Table 1:** Genetic similarity (%) between the presumed transcribed regions of the 5S rDNA of *Pseudis tocantins* and those from several anurans obtained from GenBank.

	*P*. *tocantins* type I 5S rDNA	*P*. *tocantins* type II 5S rDNA
*Anaxyrus americanus*^a^	81.36 (±4.41)	79.39 (±4.48)
*Engystomops freibergi* and *E*. *petersi* type I 5S rDNA^b^	79.03 (±4.62)	78.96 (±4.62)
*Engystomops freibergi* and *E*. *petersi* type II 5S rDNA^c^	70.83 (±5.04)	82.68 (±4.30)
*Gastrotheca riobambae*^d^	78.73 (±4.64)	82.02 (±4.23)
*Lithobates pipiens* and *L*. *catesbeianus*^e^	77.74 (±4.59)	76.75 (±4.55)
*Pelophylax lessonae* and *P*.*ridibundus*^f^	79.50 (±4.60)	80.18 (±4.52)
*Physalaemus cuvieri* type I 5S rDNA^g^	78.51 (±4.73)	79.17 (±4.60)
*Physalaemus cuvieri* type II 5S rDNA^h^	67.76 (±5.26)	82.31 (±4.20)
*Xenopus borealis*^i^	80.04 (±4.43)	74.12 (±4.85)
*Xenopus laevis* and *X*. *tropicalis* somatic 5S RNA gene^j^	77.74 (±4.60)	80.04 (±4.39)
*Xenopus laevis* and *X*. *tropicalis* oocytic 5S RNA gene^k^	75.98 (±4.61)	77.31 (±4.38)
*Pseudis tocantins* type I 5S rDNA^l^	-	64.69 (±5.41)
*Pseudis tocantins* type II 5S rDNA^m^	64.69 (±5.41)	-

Numbers in parentheses indicate the standard error. The regions corresponding to the primers 5S-A and 5S-B (used to isolate the 5S rDNA sequences of *P*. *tocantins*) were not considered in this comparative analysis.

^a^*Anaxyrus americanus* 5S rRNA gene—X58365.1 (as *Bufo americanus*)

^b^*Engystomops freibergi* type I 5S rRNA gene—JF325868.1-JF325870.1, *Engystomops petersi* type I 5S rRNA gene—JF325859.1—JF325867.1

^c^*E*. *petersi* type II 5S rRNA gene—JF325846.1-JF325858.1, *E*. *freibergi* type II 5S rRNA gene—JF325843.1-JF325845.1

^d^*Gastrotheca riobambae* 5S rRNA gene—M74438.1

^e^*Lithobates pipiens* 5S rRNA gene—X58368.1; *Lithobates catesbeianus* 5S rRNA gene—X58367.1

^f^*Pelophylax lessonae* 5S rRNA gene—FJ572051.1; *Pelophylax ridibundus* 5S rRNA gene—FJ572052.1

^g^*Physalaemus cuvieri* type I 5S rDNA—JF281126.2-JF281131.2

^h^*P*. *cuvieri* type II 5S rRNA gene—JF281132.2-JF281134.2

^i^*Xenopus* borealis 5S rRNA gene—V01425.1

^j^*Xenopus laevis* somatic 5S rRNA gene—J01009.1, *Xenopus tropicalis* somatic 5S rRNA gene—X12622.1 and NR 033271.1

^k^*X*. *laevis* oocyte specific 5S rRNA gene—J01010.1, J01012.1, M10635.1, M63899.1, X05089.1, *X*. *tropicalis* oocyte specific 5S rRNA gene -NR_033270.1, NR_033271.1, X12623.1, X12624.1

^l^sequences of type I 5S rDNA of *Pseudis tocantins* from this study

^m^sequences of type II 5S rDNA from *P*. *tocantins* from this study.

**Fig 4:**
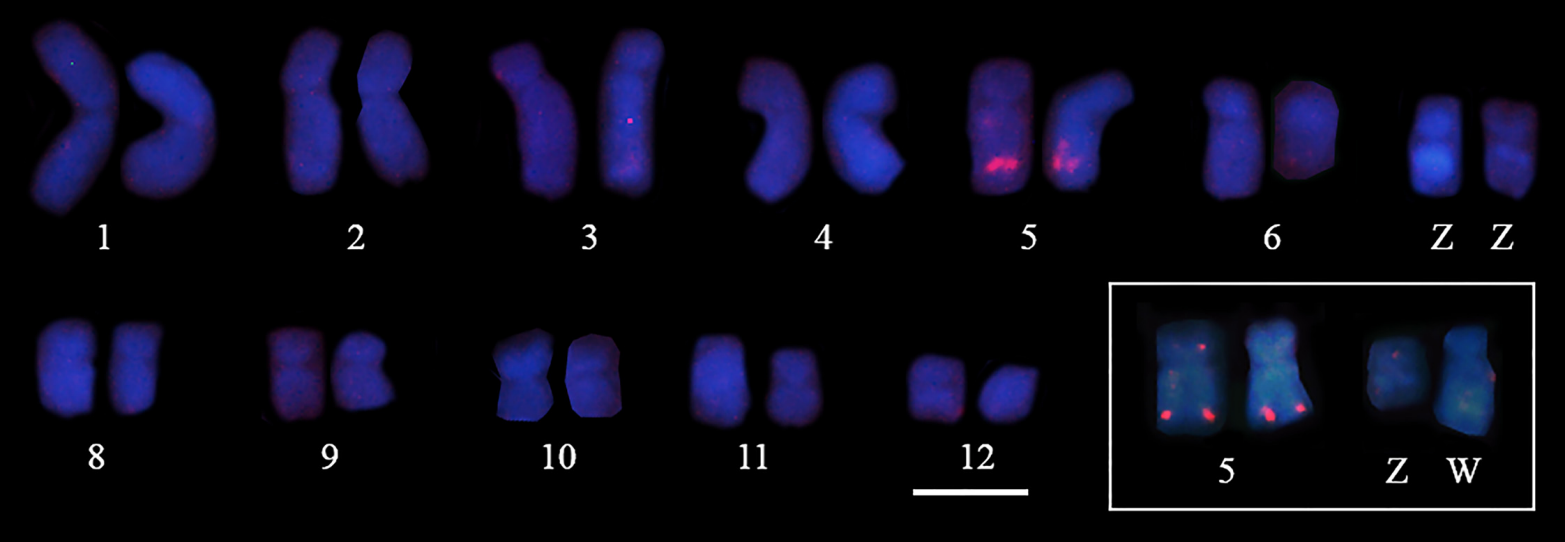
Chromosomal localization of 5S rDNA in *Pseudis tocantins*. Mapping of the type II 5S rDNA probe to a male *Pseudis tocantins* karyotype. In the inset, chromosome pair 5 and sex chromosomes of a female of *P*. *tocantins* hybridized to the 5S rDNA probe. Note that no signal of the probe is observed in the sex chromosomes. Bar: 5 μm.

When compared with the 5S rDNA sequences from other animals, including fish, chicken and human, the putative internal control region of the 5S rDNA of *Pseudis tocantins*, especially the type I sequences, showed some differences (Fig 3). The type II 5S rDNA of *P*. *tocantins* was mapped to a distal region of the long arm of chromosome 5 of males and females of this species (Fig 4), whereas the type I 5S rDNA probe did not produce any hybridization signal in our experiments.

### PcP190 sat DNA

#### Nucleotide composition

A total of 41 fragments amplified by PCR using the primers P190R and P190F were isolated and cloned. Most of them (26 inserts) contained only one partial monomer of PcP190 sat DNA, but sequences that included two (eight inserts) or three (four inserts) PcP190 repeats were also obtained. In addition, three of the cloned fragments presented complete monomers flanked by truncated PcP190 sequences. From male and female genomic DNA, six and seven fragments, respectively, were isolated and cloned, while from microdissected Z and W chromosomes, 17 and four cloned fragments were recovered, respectively (Table 2).

**Table 2:** Monomer length and similarity of the PcP190 sat DNA sequences isolated from *Pseudis tocantins* assigned to the PcP-1—PcP-7 groups.

Sequence group	Monomer length (pb)^a^	Mean similarity (%) of the more conserved region ^a^^,^ ^b^	Mean similarity (%) of the hypervariable region ^b^	Sources used to isolate the sequences
**PcP-1a**	190	?	93.42 (N = 7)	Male genomic DNA sample; microdissected W chromosome
**PcP-1b**	?	?	99.70(N = 10)	Microdissected Z chromosomes previously amplified by WGA
**PcP-2**	166–189	88.50 (N = 3)	81.31 (N = 13)	Female genomic DNA sample; male genomic DNA; microdissected Z and W chromosomes
**PcP-3**	181	?	97.73 (N = 3)	Microdissected Z and W chromosomes
**PcP-4**	173–181	90.48 (N = 3)	83.03 (N = 4)	Microdissected Z and W chromosomes
**PcP-5**	204	?	90.43 (N = 4)	Male genomic DNA sample
**PcP-6**	?	?	?	Microdissected Z chromosome
**PcP-7**	107–121	90.54 (N = 9)	-^c^	Male genomic DNA sample; microdissected Z and W chromosome

N: number of sequences compared in each analysis.

^a^Values obtained only with complete monomers.

^b^Values obtained considering the indels and only for groups with more than one sequence.

^c^Hypervariable region is absent in the PcP-7 group.

**Fig 5:**
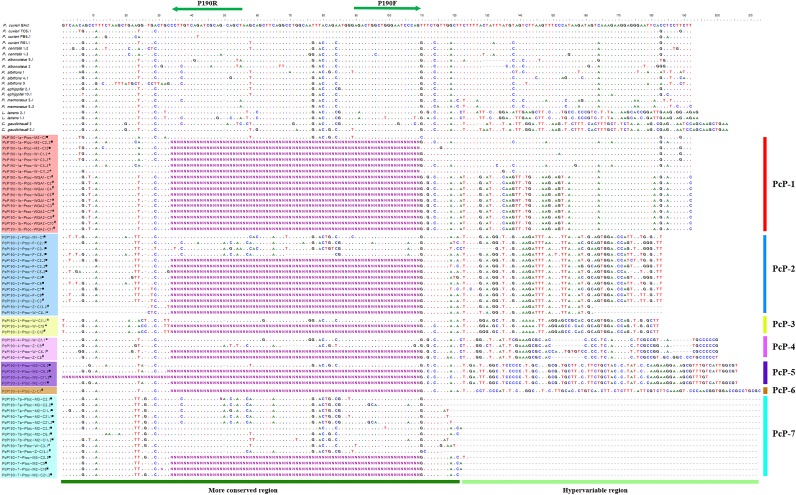
PcP190 sequences from *Pseudis tocantins* and other anurans. PcP190 sequences isolated from *Pseudis tocantins* aligned with PcP190 sequences of *Physalaemus cuvieri* (JF281121.1, KM361677.1, KM361682.1, KM361675.1), *Physalaemus centralis* (KM361684.1 and KM361685.1), *Physalaemus albonotatus* (KM361689.1 and KM361690.1), *Physalaemus albifrons* (KM361694.1, KM361696.1 and KM361698.1), *Physalaemus ephippifer* (KM361699.1 and KM361700.1), *Physalaemus marmoratus* (KM361701.1 and KM361702.1), *Leptodactylus latrans* (KM361718.1 and KM361719.1) and *Crossodactylus gaudichaudii* (KM361725.1 and KM361726.1). Annealing regions of Primer P190F and P190R are indicated with green arrows. Sequences of PcP190 from *P*. *tocantins* isolated from genomic DNA (■), microdissected W (*) and Z (#) chromosomes are indicated.

When the sequences of the PcP190 sat DNA of *Pseudis tocantins* were compared with each other and with the sequences isolated by Vittorazzi and colleagues [26, 27], two different regions could be identified, namely, a hypervariable region, which varies both in length and nucleotide composition, and a more conserved region, with an overall mean similarity of 88.41% (Fig 5; S1 Table). Based on the hypervariable region, seven different sequence groups were identified among the PcP190 sequences from *P*. *tocantins* (PcP-1 to PcP-7 sequences), with the PcP-7 sequence group being characterized by the absence of the hypervariable region (Fig 5). Sequences whose hypervariable region was highly similar (Fig 5) to the PcP190 sequences isolated from *Physalaemus* species [26, 27] were named PcP-1 sequences. Among the PcP-1 sequences of *P*. *tocantins*, two subgroups (1a and 1b) were recognized based on a differential segment at the beginning of the hypervariable region (sites 122–155 in Fig 5), such that the hypervariable region of the PcP-1a sequences of *P*. *tocantins* was 88% similar to that of the PcP-1 sequences of *Physalaemus* spp., whereas the mean similarity between the hypervariable region of the PcP-1b sequences and that of the PcP-1 sequences of the species of *Physalaemus* was 63%.

**Fig 6:**
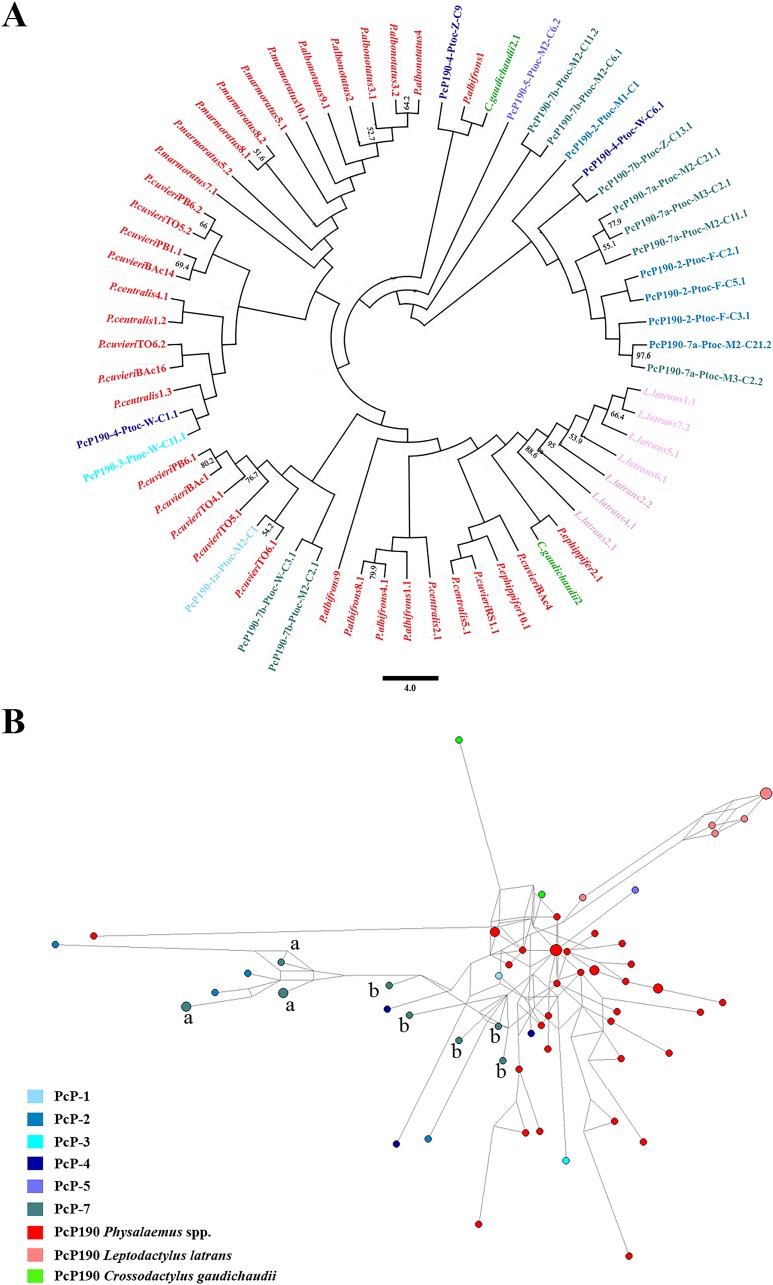
**Dendrogram from the maximum likelihood analysis (A) and neighbor-joining network (B) of the more conserved region of the PcP190 sat DNA.** Only complete monomer sequences were considered. The PcP-2 to PcP-5 and PcP-7 sequence groups of *Pseudis tocantins*, which were recognized according to their hypervariable region (see the text for details), are indicated. In *Physalaemus* spp. (red), *Leptodactylus latrans* (salmon) and *Crossodactylus gaudichaudii* (green) sequences are also included.

The classification of the PcP190 sequences in seven groups could not be achieved when only the more conserved region was considered, although some variation could be observed (Fig 5). However, among the sequences belonging to the PcP-7 group, which have no hypervariable region, two subtypes of sequences (7a and 7b) could be recognized (Fig 5). In the maximum likelihood analysis of the more conserved region of the PcP190 sequences, the subtype PcP-7a clustered with the PcP-2 sequences (Fig 6A). The network analysis also suggested a close relationship of the PcP-7a sequences with the PcP-2 sequences, whereas the PcP-7b sequences were more related to the remaining sequences, including those of *Physalaemus* (Fig 6B).

**Fig 7:**
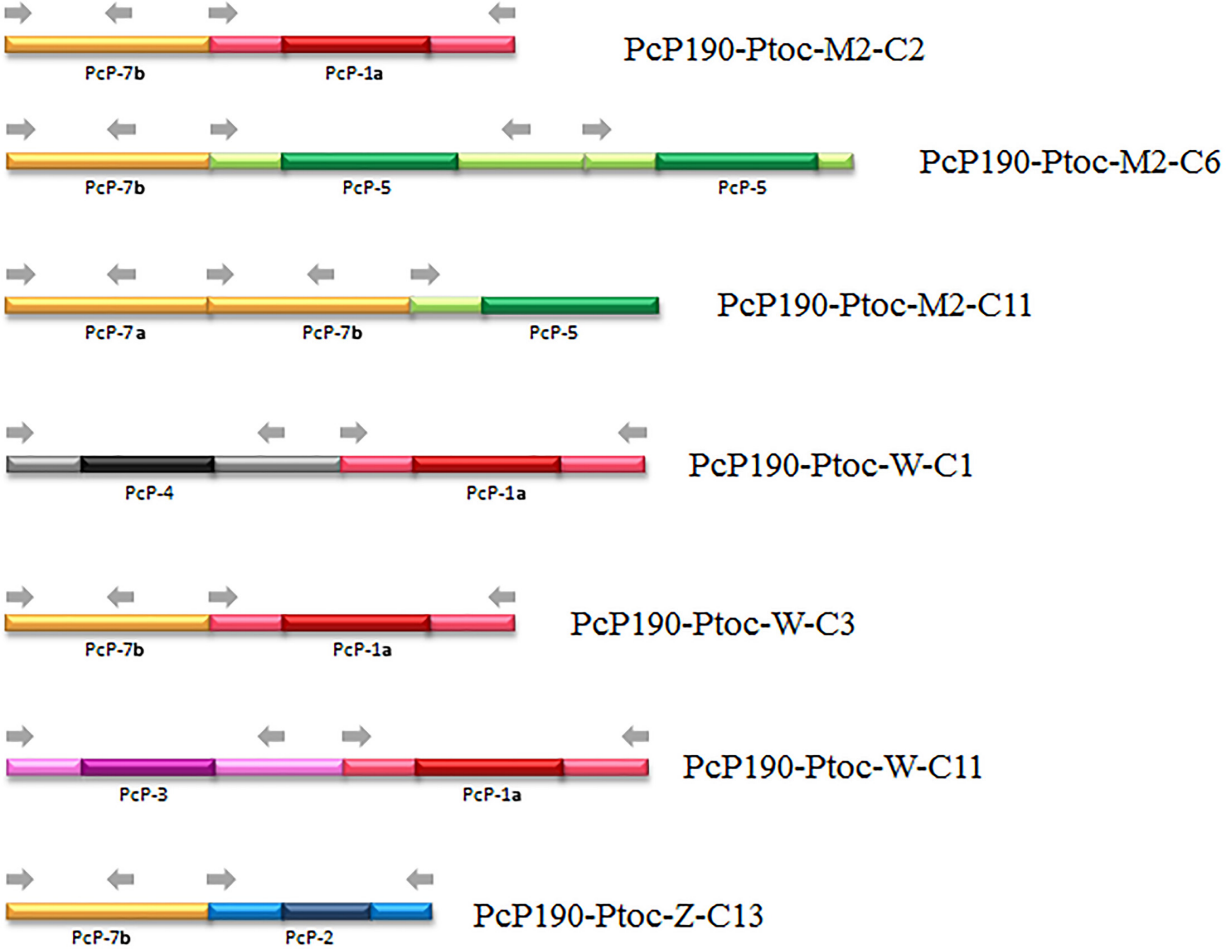
Scheme of the fragments obtained from male genomic DNA and microdissected Z and W chromosomes composed of different juxtaposed types of sequences of PcP190 sat DNA. Gray arrows indicate the annealing sites of the primers P190F and P190R used to obtain the sequences. The different colors represent different sequence types. Darker colors distinguish the hypervariable regions from the more conserved regions (light green, light gray, light red and light purple).

Another remarkable finding emerged from the analysis of the cloned fragments that included more than one PcP190 monomeric unit. Among the 12 multimeric cloned fragments, seven were composed of sequences assigned to different groups, as shown in Fig 7.

**Fig 8:**
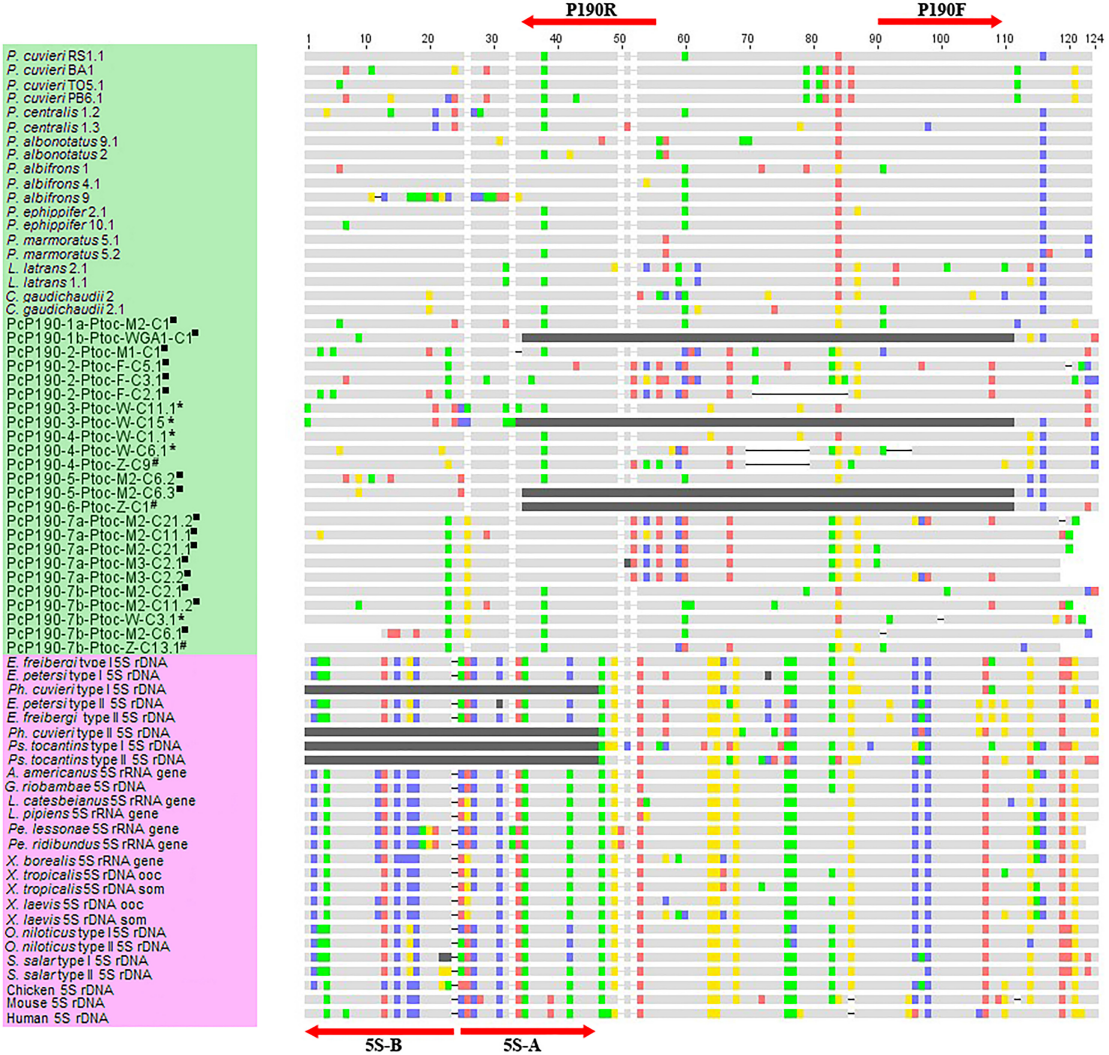
Comparison between the more conserved region of the PcP190 sequences and the transcribing region of 5S rDNA sequences. Alignment of the more conserved region of the PcP190 sequences from species of *Physalaemus* (JF281121.1, JF281117.1, JF281124.1, JF281119.1 and KM361675.1 to KM361706.1), *Leptodactylus latrans* (KM361718.1 to KM361724.1), *Crossodactylus gaudichaudii* (KM361725.1 and KM361726.1) and *Pseudis tocantins* (green shaded names) with 5S rDNA transcribing region of several vertebrate species (pink shaded names. J01009.1, J01010.1, X12622.1, X12623.1, V01425.1, M74438.1, X58368.1, X58367.1, X58365.1, JF325862.1, JF325870.1, JF325847.1, JF325845.1, JF281131.2, JF281131.2, K02235.1, X01309.1, S73106.1, S73107.1, AF478461.1, AF478462.1, K02217.1). Light gray shadows identical sequences. Dark gray represents missing data. Annealing sites of the primers commonly used to isolate these sequences are indicated. Sequences of PcP190 from *P*. *tocantins* isolated from genomic DNA (■), microdissected W (*) and Z (#) chromosomes are indicated.

Comparison of the PcP190 sequences with several 5S rDNA sequences available in GenBank revealed a noticeable correspondence between the more conserved region of the PcP190 sequences and the 5S rDNA transcribing region (*i*.*e*., the 5S rRNA gene) (Fig 8). When only the 5S rRNA genes of anurans were compared with the conserved region of the PcP190 sequences, the similarity values ranged from 56.56% to 72.37% (S2 Table) (mean similarity = 66.96%). The last 45 bp of the more conserved region of PcP190 sat DNA showed an overall mean similarity of 72.27% with the coincident region of the 5S rRNA gene. In contrast, the hypervariable region of the PcP sat DNA known to date shared no similarity with any of the non-transcribed spacer (NTS) of the 5S rDNA reported in the literature.

#### Chromosome mapping

**Fig 9:**
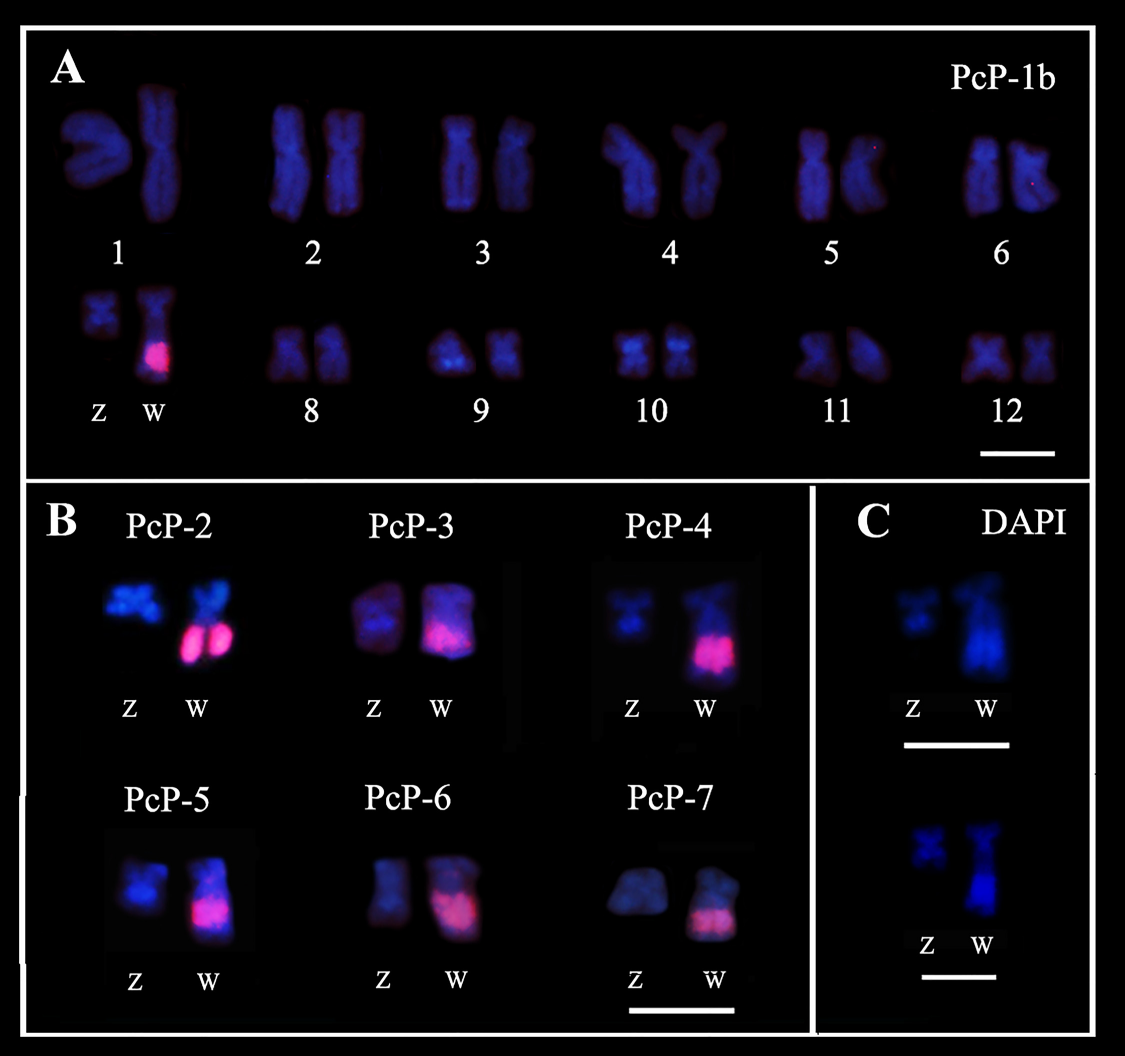
Mapping of PcP sequences to female chromosomes of *Pseudis tocantins*. (A) Fluorescent *in situ* hybridization of a probe for the PcP-1b to a female karyotype of *P*. *tocantins*. (B) Fluorescent *in situ* hybridization of probes for PcP-2, PcP-3, PcP-4, PcP-5, PcP-6 and PcP-7 sequences to ZW pairs of *P*. *tocantins*. (C) DAPI image of the ZW pairs hybrized to PcP-1b and PcP-4 probes in (A) and (B), respectively. Bar: 5 μm.

In FISH experiments, probes for the PcP-1b, PcP-2, PcP-3, PcP-4, PcP-5, PcP-6 and PcP-7 sequences detected the heterochromatic block on the long arm of the W chromosome of *Pseudis tocantins* (Fig 9). No other chromosomal segment was detected with these probes. FISH experiments with PcP-1a probes did not detect any hybridization signals in the karyotype of *P*. *tocantins*, despite they detected several centromeric/pericentromeric regions in the karyotype of *Physalaemus* aff. *cuvieri* (data not shown).

#### Southern blotting

**Fig 10:**
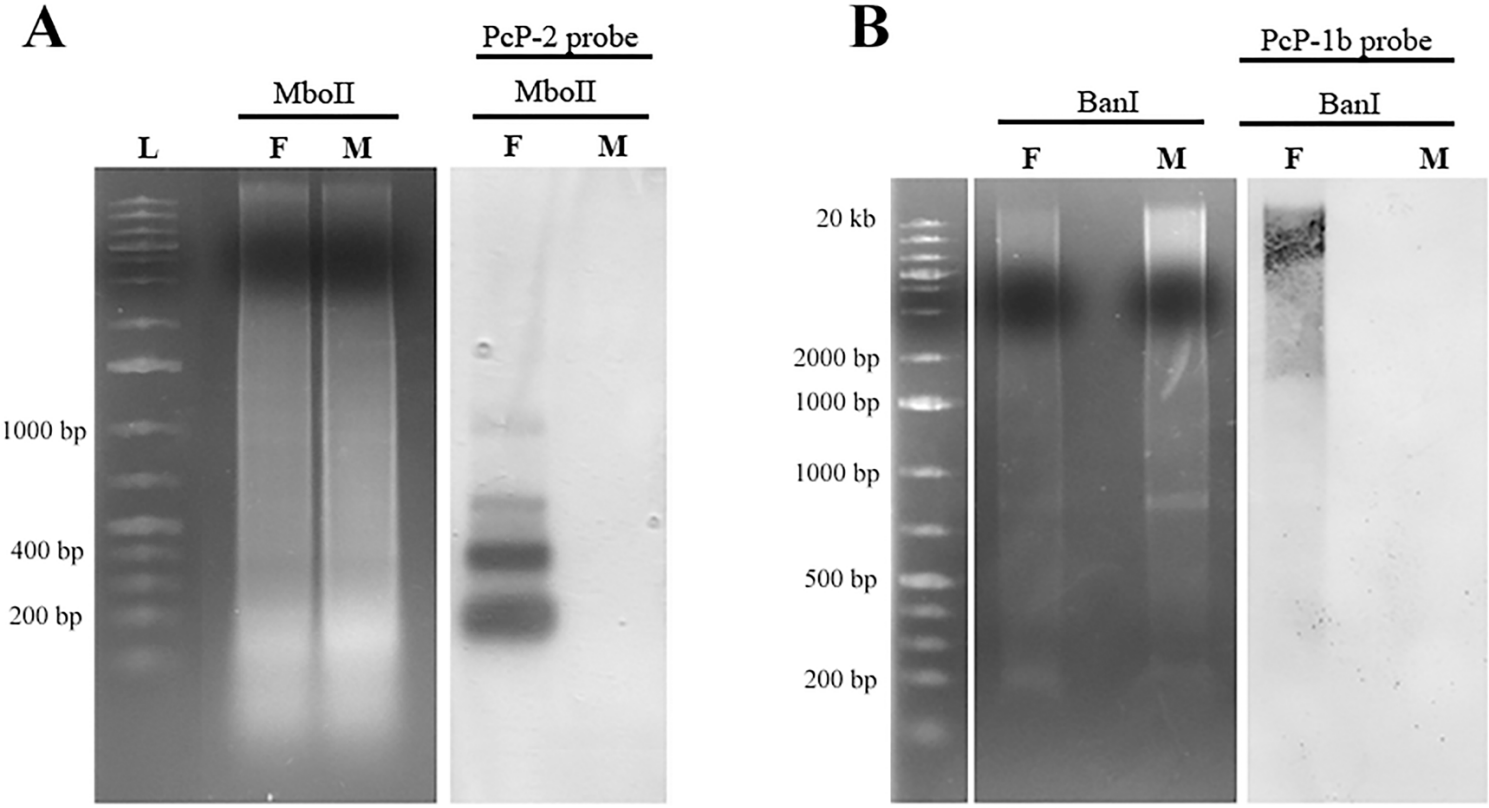
**Detection of PcP-2 (A) and PcP-1b (B) sequences of *Pseudis tocantins* by Southern blot.** (A) Samples of female (F) and male (M) genomic DNA digested with MboII and hybridized with a probe for the PcP-2 sequences. Note the detection of multiple bands only in the female sample. (B) Samples of female (F) and male (M) genomic DNA digested with BanI and hybridized with a probe for PcP-1b sequences. Note the hybridization signal on a smear of fragments with more than 1000 bp.

In the Southern blotting experiments, the female genomic DNA of *Pseudis tocantins*, digested with MboII and hybridized with a probe for PcP-2 sequence showed a ladder pattern typically found for tandemly repeated sequences (Fig 10A). In male genomic DNA digested with the same restriction endonuclease, no hybridization signal of this probe was observed (Fig 10A). When female genomic DNA was digested with BanI restriction enzymes, which cut PcP-1b sequences, a smear of high molecular weight fragments was revealed using the PcP-1b (Fig 10B) sequences as probe, suggesting that a large amount of these sequences is present but interspersed rather than organized in tandem. In contrast, male genomic DNA digested with the same enzyme and hybridized with a PcP-1b probe did not show any band in the Southern blotting (Fig 10B).

## Discussion

### 5S ribosomal DNA

Two types of 5S rDNA, easily differentiated by their presumed NTS regions, were isolated from genomic DNA of *Pseudis tocantins*. In contrast to the NTS, the presumed transcribing regions of the type I and type II sequences were comparable, despite that they were only 65% similar. As already verified for other anurans [38], the presumed transcribed region of the type I and type II 5S rDNA of *P*. *tocantins* were more highly similar with the transcribed regions of other anurans than with each other.

The presumed internal control region of the type I 5S rDNA from *Pseudis tocantins* presents low similarity with that of other species, especially with respect to the A box, raising doubt about its functionality. Because FISH experiments did not produce any signal of hybridization, it is likely that the type I 5S rDNA occurs in low copy number in the genome of *P*. *tocantins*.

There are few studies with amphibians in which the 5S rDNA clusters were cytogenetically mapped, despite the usefulness of this technique for karyotypic comparison. In *Xenopus laevis*, *X*. *borealis* [39], *Lithobates catesbianus* (*Rana catesbiana* in [40]) and *Strabomantis bipocartus* (*Eleutherodactylus maussi* in [41]), the 5S rDNA clusters are localized to terminal chromosomal regions. In the *Pseudis tocantins* karyotype the localization of type II 5S rDNA were revealed by FISH in a distal region of the long arm of chromosome 5. In *Physalaemus cuvieri* [27], *Engystomops freibergi* and *E*. *petersi* [38], the type II 5S rDNA sequences occur in a distal region of chromosome 6, but the type I 5S rDNA sequences mapped to a pericentromeric region of chromosome 3. However, in *Craugastor ranoides*, *C*. *taurus* [6], *Bombina variegata* [40], *Discoglossus pictus* and *Alytes obstericans* [42], cytogenetic mapping revealed 5S rDNA at pericentromeric or interstitial chromosomal regions.

Nakajima et al. [43] analyzed the intra-chromosomal localization of 5S rDNA in several fish species and inferred that the presence of 5S rDNA clusters at non- terminal sites might represent an ancestral condition of the 5S rRNA genes organization. For anurans, however, a proper conclusion about the prevalence and evolutionary significance of the intra-chromosomal localization of the 5S rDNA still depends on the study of a greater number of species, which could better represent all of the phylogenetic groups of this order of Amphibia.

### Sex chromosomes

Since the study of Ohno [44], the sex chromosomes have been thought to arise from ancestral homomorphic autosomal chromosomes by suppression of meiotic recombination, which may be achieved by chromosome rearrangements, such as inversions. In anurans, evidence of the occurrence of chromosomal inversion in sex chromosomes was found in *Glandirana rugosa* (*Rana rugosa* in [45–48]), *Tomopterna dellalandi* [49] and, according to Busin et al. [29], also in *Pseudis tocantins*. The relative position of the NOR and the non-centromeric heterochromatic block in the long arm of the W chromosome of *P*. *tocantins* differs from that observed in the Z chromosome of this species and also for the chromosome 7 (possible homologous to the Z chromosomes of *P*. *tocantins*) of the karyotypes of *P*. *fusca*, *P*. *bolbodactyla*, *P*. *paradoxa* and *P*. *platensis* [29], suggesting the involvement of paracentric inversion in the differentiation of the sex chromosomes found in *P*. *tocantins*.

Heterochromatin accumulation/amplification is another important phenomenon associated with morphological differentiation of the sex chromosomes in a number of organisms [19, 22, 44, 50–53]. According to Singh et al. [22] and Singh et al. [53], the accumulation of repetitive DNA segments in heterochromatin sites on W or Y chromosomes causes an asynchrony in the replication pattern of the two homologues and reduces the frequency of recombination between them. In anurans, the differential accumulation of heterochromatin between sex chromosomes was already observed in *Proceratophrys boiei* [54], *Pristimantis euphronides* and *P*. *shrevei* [55], species with W chromosomes enriched with heterochromatin, and in *Gastrotheca riobambae* [56], in which an accumulation of heterochromatin is observed in the Y chromosome. However, the differential loss of heterochromatin may also be involved in sex chromosome differentiation, as shown in anurans of the genus *Eupsophus* by Iturra and Veloso [9] and Cuevas and Formas [57]. In *Pseudis tocantins*, Busin et al. [29] showed a difference in the size of the heterochromatic bands in the long arm of the Z and W chromosomes, suggesting an amplification process of the heterochromatic block in the long arm of the W chromosome. Employing CGH experiments and the mapping of a PcP190 repetitive sequence, we could infer that the heterochromatic block of the long arm of the Z chromosome differs from that present on the long arm of the W chromosome of this species not only in size but also in composition.

CGH experiments with *Pseudis tocantins* revealed strong hybridization signals of the female genome DNA probe at the heterochromatin region of the long arm of the W chromosome, suggesting that this heterochromatin is distinct from the heterochromatic block present on the long arm of the Z chromosome. Based only on CGH, however, we cannot conclude whether the difference between the heterochromatic blocks of the Z and W chromosomes of *Pseudis tocantins* is due to the presence of distinct types of sequences or just due to a copy number variation of sequences present on both chromosomes. In addition, the study of the PcP190 satellite DNA provided further information about this issue.

The heterochromatin of the W chromosome of *Pseudis tocantins* appeared to be enriched for PcP190 sat DNA sequences, as shown by FISH with PcP-1b and PcP-2 to -7 probes. Southern blotting suggested that, in female genome, the PcP-2 sequences are tandemly repeated, whereas the PcP-1b sequences are interspersed. In contrast, Southern blotting was not able to detect the PcP-1b and PcP-2 sequences in male genome of *P*. *tocantins* and FISH did not detect any hybridization signals of the PcP-1b and PcP-2 probes in the Z chromosome, although we isolated these PcP sequences from male genomic DNA and microdissected Z chromosomes by PCR using specific primers for the PcP190 sequences. Accordingly, we conclude that the PcP-1b and PcP-2 sequences are not W-specific sequences, but are abundant in the W chromosome, and are present in a very low copy number in male genome of *P*. *tocantins*. In addition, the absence of hybridization signals of the PcP-1a probe in the karyotype of *P*. *tocantins* in FISH assays suggested that this kind of PcP sequence, in contrast to the remaining PcP sequences, is not amplified in the heterochromatin of Wq.

### PcP190 satellite DNA

Although the families of sat DNA usually present a species-specific nature [58], some of them may be present in closely related species (e.g., [59, 60]). A few ancient sat DNAs are present in several and phylogenetic distantly related taxa, such as the PstI family in sturgeons [61] and the BIV160 family in bivalve mussels [62]. The PcP190 sat DNA was previously detected in anurans allocated in Leptodactylidae and Hylodidae [26], and based on the divergence time estimated by Fouquet et al. [63] for these taxonomic families, Vittorazzi et al. [26] inferred that the PcP190 sat DNA originated approximately 70 million years ago. The existence of the PcP190 sat DNA in *Pseudis tocantins*, which is an anuran allocated in the family Hylidae, suggest that this sat DNA could be more ancient than inferred by Vittorazzi et al. [26] because Hylidae occupies a more basal position in Hyloidea when compared with Leptodactylidae and Hylodidae [63, 64].

A genus-specific pattern for the PcP190 sat DNA was proposed by Vittorazzi et al. [26] after the identification of a region with approximately 74 bp that differed among the three genera analyzed (*Physalaemus*, *Leptodactylus* and *Crossodactylus*,), but that was typical for each of them. In our analysis, a hypervariable region could also be recognized among the PcP190 sequences isolated from *Pseudis tocantins*, allowing their classification in seven groups. The great similarity found between the *P*. *tocantins* sequences included in the PcP-1 group and the sequences from *Physalaemus* spp. reject the hypothesis that the hypervariable region could be genus-specific. In addition, the high similarity between such sequences, and found in distantly related species, allowed us to consider this sequence as an ancestral sequence of the PcP190 sat DNA.

In general, sequence homogeneity among the monomers of a sat DNA family is expected because repetitive DNA sequences may evolve in concert by a process called molecular drive [65–67]. The occurrence of different groups of PcP190 sequences in the genome of *Pseudis tocantins* suggests that the homogenization process may not be as effective for this sat DNA, although the finding of interspersed arrangements of sequences assigned to different groups suggests that these sequence groups are not clustered apart in exclusive chromosome sites. Interspersed arrangements of different types of monomers as those found here were previously reported for some sat DNA families, like the pBuM and DBC-150 sat DNAs found in species of *Drosophila* [60, 68].

In addition, the existence of distinct subtypes among the PcP190 sequences that lack the hypervariable region (PcP-7a and PcP7b sequences) may provide some evidence of recurrent rearrangements involving the PcP190 sequences. The high similarity of the PcP-7a sequences with the PcP-2 sequences, and the similarity between the PcP-7b sequences and PcP-1 sequences (and consequently the PcP190 sequences from *Physalaemus* spp.) may be explained by the independent origin of the PcP-7a and PcP-7b sequences from the PcP-2 and PcP-1 sequences, respectively, and by the deletion of hypervariable region. On the other hand, an alternative hypothesis may also explain our findings, according to which the PcP-7a and PcP-7b sequences would share the evolutionary event that resulted in the loss of the hypervariable region, originating an ancestral PcP-7 sequence. In this case, the differentiation of the PcP-7 group into two subtypes could be achieved, subsequently, by recombination between conserved regions.

Another important question is raised from the comparison of the PcP190 sat DNA and 5S rDNA. Approximately, 120 bp of the repetitive unit of the PcP190 sat DNA are very similar to the transcribing region of 5S rDNA sequences, a fact that supported Vittorazzi et al. (2011) to infer the origin of this sat DNA from 5S rDNA. In contrast, the remaining 60–91 bp of the PcP190 repetitive units constitute a hypervariable region among the PcP190 sequences, which are not similar with any of the NTS sequence of 5S rDNA reported to date. Two hypotheses could explain such a differential pattern of variation in the PcP190 sat DNA. The first hypothesis is the recurrent occurrence of illegitimate recombination events between PcP190 sat DNA and variants of the 5S rDNA. Illegitimate recombination was previously invoked by Kuhn et al. [68] to explain a variety of junctions observed between two non-homologous sat DNAs (pBuM and DBC-15) in *Drosophila buzzatti* cluster species.

In eukaryotes, recombination depends on both the length and identity of the sequences involved in the event. Rubnitz and Subramani [69], based on plasmid transfection assay in mammalian cell lines, reported that the recombination frequency declines as the sequence length decreases, and they found that the minimum length for efficient recombination at high frequencies of identical sequences is 200 bp. However, the same authors and Ayares et al. [70] found a low recombination frequency even between sequences with only 25 bp. In another important study, Opperman et al. [71] showed that 0.16% of divergence (one mutation in a sequence with 618 bp) decreased the recombination rate approximately threefold, although recombination events still occur. Okumura et al. [72], based on human α-satellite, observed that recombination events could occur among heterologous subunits with 70–80% similarity.

The corresponding regions between the PcP190 repeats and the anuran 5S rRNA genes are extended by approximately 120 bp and show 59.70% to 70.83% similarity. A higher level of similarity was found (72%) if only the last 45 bp of these corresponding regions are compared. Therefore, eventual recombination between PcP190 sat DNA and 5S rDNA may have occurred during the evolution of these repetitive families, which could have carried NTS from different 5S rDNA sequences to the PcP190 sat DNA. As a result, a hypervariable region would have arisen in the PcP190 sat DNA that originated from different NTS sequences of 5S rDNA.

Alternatively, the lower sequence variation in a specific region of the PcP190 sat DNA repeats could be explained by a differential selective pressure, as suggested previously [26]. Vittorazzi et al. [26, 27] mapped the PcP190 sat DNA in centromeric/pericentromeric regions of several *Physalaemus* species, and raised the hypothesis that this sat DNA could be involved in centromere biology in the studied species. In *Pseudis tocantins*, the centromeres are not enriched with PcP190 sat DNA sequences and the PcP-1a sequence, which is more similar with the PcP190 from *Physalaemus* species, is in a low copy number, as inferred from the FISH experiments. However, the PcP-1b and PcP-2 sequences are abundant in the heterochromatic block on Wq, and despite not being detectable by FISH, this type of sequence is also present in other sites of the genome, even in the Z chromosome, as revealed the analysis of microdissected chromosomes. Therefore, in *P*. *tocantins* the PcP190 sat DNA may have played a role in the evolutionary differentiation of the sex chromosomes, and may have a function in the heterochromatin of the W chromosome.

The differential distribution of PcP190 sequences in heterochromatic bands of the sex chromosomes had been previously detected in *Physalaemus ephippifer* by Vittorazzi et al. (2014), but with respect to a smaller band than that observed in the W chromosome of *Pseudis tocantins*, and in addition to its pericentromeric occurrence in one autosome pair (pair 3). Divergences of repetitive DNA sequences in sex chromosomes have been documented in a number of species. Nakayama et al. [51], for example, isolated two W-specific repetitive sequences in the fish *Leporinus elongatus*. Mariotti et al. [21] described an accumulation of a specific satellite DNA on the Y chromosome of *Rumex acetosa*. Such differences may result from the accelerated molecular differentiation observed in the sex chromosomes caused by the suppression of recombination (reviewed in [3]). On the other hand, Lepesant et al. [73] hypothesized that sat DNA could play a role in the sex determination in *Schistosoma mansoni*. The authors found that specific sat DNA in the W chromosome of *S*. *mansoni* transcribes non-coding RNAs that may be involved in the chromatin compaction on the W chromosome, and inferred that these structural changes interfere with the transcription of gene(s), which may result in the development of the male and female phenotypes. According to the hypothesis of Lepessant and colleagues [73], the changes in chromatin structure induced by sat DNA could actually be the initial event in sex chromosome origin, adding new importance to investigations of this kind of sequences.

## Conclusions

The PcP190 sat DNA was shown to be ancient in the Hyloidea superfamily of Anura because it can be found not only in Leptodactylidae and Hylodidae but also in Hylidae.

The existence of a hypervariable and a more conserved region among the PcP190 sequences may be explained by illegitimate recombination with 5S rDNA, although a high selective pressure derived from a hypothetical function of the more conserved region could not be excluded.

The accumulation of PcP sequences in the heterochromatin of the W chromosome of *Pseudis tocantins* suggests that the PcP190 sat DNA may have played a relevant role in the process of sex chromosome differentiation in this species.

## Supporting Information

S1 Table Mean similarity (%) and standard error (±) between the more conserved region of the PcP190 sequences of *Pseudis tocantins*, *Physalaemus cuvieri*, *Physalaemus centralis*, *Physalaemus albonotatus*, *Physalaemus albifrons*, *Physalaemus ephippifer*, *Physalaemus marmoratus*, *Leptodactylus latrans* and *Crossodactylus gaudichaudii*. (PDF)

S2 Table Genetic similarity (%) between the presumed transcribed region of 5S rDNA from several anurans and the more conserved region of the PcP190 sequences of *Physalaemus*, *Leptodactylus*, *Crossodactylus* and *Pseudis*. (PDF)

## References

[1] Ellegren H. Sex-chromosome evolution: recent progress and the influence of male and female heterogamety. Nat Rev Genet 2011;12: 157–166. 10.1038/nrg2948 21301475

[2] Valenzuela N. Sexual development and the evolution of sex determination. Sex Dev 2008;2: 64–72. 10.1159/000129691 18577873

[3] Graves JAM. Weird animal genomes and the evolution of vertebrate sex and sex chromosomes. Annu Rev Genet 2008;42: 565–586. 10.1146/annurev.genet.42.110807.091714 18983263

[4] Schmid M, Steinlein C, Bogart JP, Feichtinger W, Haaf T, Nanda I, et al The hemiphractid frogs: phylogeny, embriology, life history and cytogenetics. Cytogenet Genome Res 2012;138: 68–384. 10.1159/000343460 23429349

[5] Schmid M, Steinlein C, Feichtinger W, Bogart JP. Chromosome banding in Amphibia. XXXI. The neotropical anuran families Centrolenidae and Allophrynidae. Cytogenet Genome Res 2014;142: 268–285. 10.1159/000362216 24776617

[6] Schmid M, Steinlein C, Bogart JP, Feichtinger W, León P, La Marca E, et al The chromosomes of terraranan frogs. Insights into vertebrate cytogenetics. Cytogenet Genome Res 2010;130–131: 1–568. 10.1159/000301339 21063086

[7] Green DM. Cytogenetics of the endemic New Zealand frog, *Leiopelma hochstetteri*: extraordinary supernumerary chromosome variation and a unique sex-chromosome system. Chromosoma 1988;97: 55–70.

[8] Hillis DM, Green DM. Evolutionary phylogenetic changes of heterogametic history of amphibians. J Evol Biol 1990;64: 49–64.

[9] Iturra P, Veloso A. Further evidence for early sex chromosome differentiation of anuran species. Genetica 1989;78: 25–31. 10.1007/BF00058671 3248710

[10] Schmid M, Ohta S, Steinlein C, Guttenbach M. Chromosome banding in Amphibia XIX. Primitive ZW/ZZ sex chromosomes in *Buergeria buergeri* (Anura, Rhacophoridae). Cytogenet Cell Genet 1993;62: 238–246. 8440144 10.1159/000133486

[11] Abramyan J, Ezaz T, Graves JAM, Koopman P. Z and W sex chromosomes 536 in the cane toad (*Bufo marinus*). Chromosome Res 2009;17: 1015–1024. 10.1007/s10577-009-9095-1 19936947

[12] Miura I, Ezaz T, Ohtani H, Uno Y, Nishida C, Matsuda Y et al 2009. The W chromosome evolution and sex-linked genes expression in the Japanese frog *Rana rugosa* In: Weingarten C, Jefferson CC, editors. Sex Chromosomes: Genetics, Abnormalities and Disorders. New York: Nova Science Publishers, Inc; 2009 pp. 123–140.

[13] Uno Y, Nishida C, Takagi C, Igawa T, Ueno N, Sumida M, et al Extraordinary diversity in the origins of sex chromosomes in anurans inferred from comparative gene mapping. Cytogenet Genome Res 2015;145: 218–229. 10.1159/000431211 26089094

[14] Koubová M, Pokorná MJ, Rovatsos M, Farkačová K, Altmanová M, Kratochvíl L. Sex determination in Madagascar geckos of the genus *Paroedura* (Squamata: Gekkonidae): are differentiated sex chromosomes indeed so evolutionary stable? Chromosome Res 2014;22: 441–452. 10.1007/s10577-014-9430-z 25056523

[15] Matsubara K, Sarre SD, Georges A, Matsuda Y, Graves JAM, Ezaz T. Highly differentiated ZW sex microchromosomes in the australian varanus species evolved through rapid amplification of repetitive sequences. PLoS One 2014;9(4). 10.1371/journal.pone.0095226 PMC3990592 24743344

[16] Vítková M, Fuková I, Kubíčková S, Marec F. Molecular divergence of the W chromosomes in pyralid moths (Lepidoptera). Chromosome Res 2007;15: 917–930. 17985203 10.1007/s10577-007-1173-7

[17] Ayling LJ, Griffin DK. The evolution of sex chromosomes. Cytogenet. Genome Res 2002;99: 125–140. 12900555 10.1159/000071584

[18] da Silva EL, Busso AF, Parise-Maltempi PP. Characterization and genome organization of a repetitive element associated with the nucleolus organizer region in *Leporinus elongatus* Anostomidae: Characiformes). Cytogenet. Genome Res 2013;139: 22–28. 10.1159/000342957 23037972

[19] Hobza R, Lengerova M, Svoboda J, Kubekova H, Kejnovsky E, Vyskot B. An accumulation of tandem DNA repeats on the Y chromosome in *Silene latifolia* during early stages of sex chromosome evolution. Chromosoma 2006;115: 376–382. 16612641 10.1007/s00412-006-0065-5

[20] Itoh Y, Mizuno S. Molecular and cytological characterization of SspI-family repetitive sequence on the chicken W chromosome. Chromosome Res 2002;10: 499–511. 12489831 10.1023/a:1020944414750

[21] Mariotti B, Manzano S, Kejnovský E, Vyskot B, Jamilena M. Accumulation of Y-specific satellite DNAs during the evolution of *Rumex acetosa* sex chromosomes. Mol Genet Genomics 2009;281: 249–259. 10.1007/s00438-008-0405-7 19085011

[22] Singh L, Purdom F, Jones KW. Satellite DNA and evolution of sex chromosomes. Chromosoma 1976;62: 43–62. 10.1007/BF00327708 1001165

[23] Amor N, Odierna G, Chinali G, Said K, Picariello O. Unusual chromosomal distribution of a major satellite DNA from Discoglossus pictus (Amphibia, Anura). Cytogenet. Genome Res 2009;127: 33–42. 10.1159/000279444 20110657

[24] Odierna G, Aprea G, Capriglione T, Castellano S, Balletto E. Evidence for chromosome and Pst I satellite DNA family evolutionary stasis in the *Bufo viridis* group (Amphibia, Anura). Chromosome Res 2004;12: 671–81. 15505402 10.1023/B:CHRO.0000045746.59805.58

[25] Ragghianti M, Guerrini F, Bucci S, Mancino G, Hotz H, Uzzell T, et al Molecular characterization of a centromeric satellite DNA in the hemiclonal hybrid frog *Rana esculenta* and its parental species. Chromosome Res 1995;3: 497–506. 8581303 10.1007/BF00713965

[26] Vittorazzi S, Lourenço LB, Recco-Pimentel S. Long-time evolution and highly dynamic satellite DNA in leptodactylid and hylodid frogs. BMC Genet 2014;15: 111 10.1186/s12863-014-0111-x 25316286 PMC4201667

[27] Vittorazzi SE, Lourenço LB, Del-Grande ML, Recco-Pimentel SM. Satellite DNA derived from 5S rDNA in *Physalaemus cuvieri* (Anura, Leiuperidae). Cytogenet Genome Res 2011;134: 101–107. 10.1159/000325540 21464559

[28] Caramaschi U, Cruz CAG. Notas taxonômicas sobre *Pseudis fusca* Garman e *P*. *bolbodactyla* A. Lutz, com a descrição de uma nova espécie correlata (Anura, Pseudidae). Rev Bras Zool 1998;15: 929–944.

[29] Busin CS, Andrade GV, Bertoldo J, Del Grande ML, Uetanabaro M, Recco- Pimentel SM. Cytogenetic analysis of four species of *Pseudis* (Anura, Hylidae), with the description of ZZ/ZW sex chromosomes in *P*. *tocantins*. Genetica 2008;133: 119–127. 17713858 10.1007/s10709-007-9189-7

[30] Medeiros LR, Lourenço LB, Rossa-Feres DC, Lima AP, Andrade GV, Giaretta AA, et al Comparative cytogenetic analysis of some species of the *Dendropsophus microcephalus* group (Anura, Hylidae) in the light of phylogenetic inferences. BMC Genet 2013;14: 59 10.1186/1471-2156-14-59 23822759 PMC3710474

[31] Pendas AM, Moran P, Freije JP, Garcia-Vazquez E. Chromosomal mapping and nucleotide sequence of two tandem repeats of Atlantic salmon 5S rDNA. Cytogenet Cell Genet 1994;67: 31–36. 8187548 10.1159/000133792

[32] Sambrook J, Russel DW. Molecular Cloning: A Laboratory Manual. New York: Cold Spring Harbor Laboratory Press; 2001.

[33] Hall TA. BioEdit: a user-friendly biological sequence alignment editor and analysis program for Windows 95/98/NT. Nucleic Acids Symp Ser 1999;41: 95–98.

[34] Tamura K, Stecher G, Peterson D, Filipski A, Kumar S. MEGA6: Molecular evolutionary genetics analysis—Version 6.0. Mol Biol Evol 2013;30: 2725–2729. 10.1093/molbev/mst197 24132122 PMC3840312

[35] Bandelt H-J, Forster P, Röhl A. Median-Joining networks for inferring intraspecific phylogenies. Mol Biol Evol 1994;16: 37–48. 10.1093/oxfordjournals.molbev.a026036 10331250

[36] Librado P, Rozas J. DnaSP v5: A software for comprehensive analysis of DNA polymorphism data. Bioinformatics 2009;25: 1451–1452. 10.1093/bioinformatics/btp187 19346325

[37] Viegas-Péquignot E. In situ hybridization to chromosomes with biotinylated probes In: Willernson D, editor. In Situ Hybridization: A practical approach. Oxford: Oxford University Press; 1992 pp. 137–158.

[38] Rodrigues D, Rivera M, Lourenço LB. Molecular organization and chromosomal localization of 5S rDNA in Amazonian *Engystomops* (Anura, Leiuperidae). BMC Genet 2012;13: 17 10.1186/1471-2156-13-17 22433220 PMC3342222

[39] Harper ME, Price J, Korn LJ. Chromosomal mapping of *Xenopus* 5S genes: somatic-type versus oocyte-type. Nucleic Acids Res 1983;11: 2313–2323. 6687939 10.1093/nar/11.8.2313 PMC325886

[40] Vitelli L, Batistoni R, Andronico F, Nardi I, Barsacchi-Pilone G. Chromosomal localization of 18S + 28S and 5S ribosomal RNA genes in evolutionarily diverse anuran amphibians. Chromosoma 1982;84: 475–491. 7075349 10.1007/BF00292849

[41] Schmid M, Feichtinger W, Steinlein C, Haaf T, Schartl M, Visbal García R et al Chromosome banding in Amphibia XXVI. Coexistence of homomorphic XY sex chromosomes and a derived Y-autosome translocation in *Eleutherodactylus maussi* (Anura, Leptodactylidae). Cytogenet Genome Res 2002;99: 330–343. 12900583 10.1159/000071612

[42] Schmid M, Vitelli L, Batistoni R. Chromosome banding in Amphibia XI. Constitutive heterochromatin, nucleolus organizers, 18S+28S and 5S ribosomal RNA genes in Ascaphidae, Pipidae, Discoglossidae and Pelobatidae. Chromosoma 1987;95: 271–284. 3622081 10.1007/BF00294784

[43] Nakajima RT, Cabral-de-Mello DC, Valente GT, Venere PC, Martins C. Evolutionary dynamics of rRNA gene clusters in cichlid fish. BMC Evol Biol 2012;12: 198 10.1186/1471-2148-12-198 23035959 PMC3503869

[44] Ohno S. Sex chromosomes and sex-linked genes. New York: Springer-Verlag; 1967.

[45] Miura I, Ohtani H, Hanada H, Ichikawa Y, Kashiwagi A, Nakamura M. Evidence for two successive pericentric inversions in sex lampbrush chromosomes of *Rana rugosa* (Anura: Ranidae). Chromosoma 1997;106: 178–82. 9233991 10.1007/s004120050237

[46] Nishioka M, Hanada H, Miura I, Ryuzaki M. Four kinds of sex chromosomes in *Rana rugosa*. Sci Rep Lab Amphibian. Biol 1994;13: 1–34.

[47] Nishioka M, Miura I, Saitoh K. Sex Chromosomes of *Rana rugosa* with special reference to local differences in sex-determining mechanism. Sci Rep Lab Amphibian Biol 1993;12: 55–81.

[48] Uno Y, Nishida C, Oshima Y, Yokoyama S, Miura I, Matsuda Y, et al Comparative chromosome mapping of sex-linked genes and identification of sex chromosomal rearrangements in the Japanese wrinkled frog (*Rana rugosa*, Ranidae) with ZW and XY sex chromosome systems. Chromosome Res 2008;16: 637–47. 10.1007/s10577-008-1217-7 18484182

[49] Schmid M. Chromosome banding in Amphibia V. Highly differentiated ZW/ZZ sex chromosomes and exceptional genome size in *Pyxicephalus adspersus* (Anura, Ranidae). Chromosoma 1980;80: 69–96.

[50] Beçak W, Beçak ML, Nazareth HRS, Ohno S. Close karyological kinship between reptilian suborder Serpentes and the class Aves. Chromosoma 1964;15: 606–617. 14333153 10.1007/BF00319994

[51] Nakayama I, Foresti F, Tewari R, Schartl M, Chourrout D. Sex chromosome polymorphism and heterogametic males revealed by two cloned DNA probes in the ZW/ZZ fish *Leporinus elongatus*. Chromosoma 1994;103: 31–39. 8013252 10.1007/BF00364723

[52] Parise-Maltempi PP, Martins C, Oliveira C, Foresti F. Identification of a new repetitive element in the sex chromosomes of *Leporinus elongatus* (Teleostei: Characiformes: Anostomidae): new insights into the sex chromosomes of *Leporinus*. Cytogenet. Genome Res 2007;116: 218–223. 17317963 10.1159/000098190

[53] Singh L, Purdom IF, Jones KW. Sex chromosome associated satellite DNA: evolution and conservation. Chromosoma 1980;79: 137–57. 7398495 10.1007/BF01175181

[54] Ananias F, Modesto ADS, Mendes SC, Napoli MF. Unusual primitive heteromorphic ZZ/ZW sex chromosomes in *Proceratophrys boiei* (Anura, Cycloramphidae, Alsodinae), with description of C-Band interpopulational polymorphism. Hereditas 2007;144: 206–212. 18031355 10.1111/j.2007.0018-0661.02026.x

[55] Schmid M, Feichtinger W, Steinlein C, Rupprecht A, Haaf T, Kaiser H. Chromosome banding in Amphibia XXIII. Giant W sex chromosomes and extremely small genomes in *Eleutherodactylus euphronides* and E*leutherodactylus shrevei* (Anura, Leptodactylidae). Cytogenet. Genome Res 2002;94: 81–94. 10.1159/000064055 12438744

[56] Schmid M, Haaf T, Geile B, Sims S. Chromosome banding in Amphibia VIII. An unusual XY/XX-sex chromosome system in *Gastrotheca riobambae* (Anura, Hylidae). Chromosoma 1983;88: 69–82. 6192977 10.1007/BF00329505

[57] Cuevas CC, Formas JR. Heteromorphic sex chromosomes in *Eupsophus insularis* (Amphibia: Anura: Leptodactylidae). Chromosome Res 1996;4: 467–70. 8889246 10.1007/BF02265054

[58] Tsoumani KT, Drosopoulou E, Mavragani-Tsipidou P, Mathiopoulos KD. Molecular characterization and chromosomal distribution of a species-specific transcribed centromeric satellite repeat from the olive fruit fly, *Bactrocera oleae*. PLoS One 2013;8(11). 10.1371/journal.pone.0079393 PMC3828357 24244494

[59] Acosta MJ, Marchal JA, Fernández-Espartero C, Romero-Fernández I, Rovatsos MT, Giagia-Athanasopoulou EB, et al Characterization of the satellite DNA Msat-160 from species of Terricola (Microtus) and Arvicola (Rodentia, Arvicolinae). Genetica 2010;138: 1085–1098. 10.1007/s10709-010-9496-2 20830505

[60] Kuhn GCS, Sene FM, Moreira-Filho O, Schwarzacher T, Heslop-Harrison JS. Sequence analysis, chromosomal distribution and long-range organization show that rapid turnover of new and old pBuM satellite DNA repeats leads to different patterns of variation in seven species of the *Drosophila buzzatii* cluster. Chromosome Res 2008;16: 307–324. 10.1007/s10577-007-1195-1 18266060

[61] Robles F, de la Herrán R, Ludwig A, Rejón CR, Rejón MR, Garrido-Ramos MA. Evolution of ancient satellite DNAs in sturgeon genomes. Gene 2004;338: 133–142. 15302414 10.1016/j.gene.2004.06.001

[62] Plohl M, Petrović V, Luchetti A, Ricci A, Satović E, Passamonti M, Mantovani B. Long-term conservation vs high sequence divergence: the case of an extraordinarily old satellite DNA in bivalve mollusks. Heredity 2010;104: 543–551. 10.1038/hdy.2009.141 19844270

[63] Fouquet A, Blotto BL, Maronna MM, Verdade VK, Juncá FA, de Sá R, Rodrigues MT. Unexpected phylogenetic positions of the genera *Rupirana* and *Crossodactylodes* reveal insights into the biogeography and reproductive evolution of leptodactylid frogs. Mol Phylogenet Evol 2013;67: 445–457. 10.1016/j.ympev.2013.02.009 23454092

[64] Pyron AR, Wiens JJ. A large-scale phylogeny of Amphibia including over 2800 species, and a revised classification of extant frogs, salamanders, and caecilians. Mol Phylogenet Evol. 2011;61: 543–583. 10.1016/j.ympev.2011.06.012 21723399

[65] Dover G. Molecular drive: a cohesive mode of species evolution. Nature 1982; 299: 111–117. 7110332 10.1038/299111a0

[66] Dover GA. Molecular drive in multigene families: how biological novelties arise, spread and are assimilated. Trends Genet 1986;168: 159–165.

[67] Plohl M, Meštrović N, Mravinac B. Satellite DNA evolution. Genome Dyn 2012;7: 126–152. 10.1159/000337122 22759817

[68] Kuhn GCS, Teo CH, Schwarzacher T, Heslop-Harrison JS. Evolutionary dynamics and sites of illegitimate recombination revealed in the interspersion and sequence junctions of two nonhomologous satellite DNAs in cactophilic *Drosophila* species. Heredity 2009;102: 453–464. 10.1038/hdy.2009.9 19259119

[69] Rubnitz J, Subramani S. The minimum amount of homology required for homologous recombination in mammalian. Mol Cell Biol 1984;4: 2253–2258. 6096689 10.1128/mcb.4.11.2253-2258.1984 PMC369052

[70] Ayares D, Chekuri L, Song KY, Kucherlapati R. Sequence homology requirements for intermolecular recombination in mammalian cells. Proc Natl Acad Sci USA 1986;83: 5199–5203. 3523485 10.1073/pnas.83.14.5199 PMC323918

[71] Opperman R, Emmanuel E, Levy AA. The effect of sequence divergence on recombination between direct repeats in *Arabidopsis*. Genetics 2004;168: 2207–2215. 15611187 10.1534/genetics.104.032896 PMC1448723

[72] Okumura K, Kiyama R, Oishi M. Sequence analyses of extrachromosomal Sau3A and related family DNA: analysis of recombination in the excision event. Nucl Acids Res 1987;15: 7477–7489. 2889188 10.1093/nar/15.18.7477 PMC306262

[73] Lepesant JM, Cosseau C, Boissier J, Freitag M, Portela J, Climent D, et al Chromatin structure changes around satellite 624 repeats on the *Schistosoma mansoni* female sex chromosome suggest a possible 625 mechanism for sex chromosome emergence. Genome Biol 2012;13: R14 10.1186/gb-2012-13-2-r14 22377319 PMC3701142

