# Supplementary material for: Unraveling the Sex Chromosome Heteromorphism of the Paradoxical Frog Pseudis tocantins
Source: PLoS One. 2016 May 23;11(5):e0156176. doi: 10.1371/journal.pone.0156176 (PMC4877019; doi:10.1371/journal.pone.0156176)
Supplement: S1 Table — (PDF) [file pone.0156176.s001.pdf]

S1 Table. Mean similarity (%) and standard error (±) between the more conserved region of the PcP190 sequences of *Pseudis tocantins*, *Physalaemus cuvieri*, *Physalaemus centralis*, *Physalaemus albonotatus*, *Physalaemus albifrons*, *Physalaemus ephippifer*, *Physalaemus marmoratus*, *Leptodactylus latrans* and *Crossodactylus gaudichaudii*. Only complete monomers were used in this analysis. Because no complete monomer of PcP-6 was achieved, this kind of sequence was not included. N: number of sequences from each group.

	P. cuvieri[a] **(n=13)**	***P. centralis***[b] **(n=5)**	***P. albonotatus***[c] **(n=5)**	***P. albifrons***[d] **(n=5)**	***P. ephippifer***[e] **(n=2)**	***P. marmoratus***[f] **(n=6)**	***L. latrans***[g] **(n=7)**	***C. gaudichaudii***[h] **(n=2)**	**PcP-1a**	**PcP-2**	**PcP-3**	**PcP-4**	**PcP-5**	**PcP-7a**
**PcP-1 (n=1)**	90.31 (±2.08)	90.83 (±2.18)	88.17 (±2.67)	89.63 (±2.23)	92.50 (±2.30)	89.99 (±2.52)	83.33 (±3.12)	88.75 (±2.44)	-					
**PcP-2 (n=4)**	81.86 (±2.54)	82.12 (±2.61)	82.98 (±2.63)	83.34 (±2.41)	85.46 (±2.55)	85.05 (±2.48)	80.80 (±2.98)	83.01 (±2.66)	82.28 (±2.91)	-				
**PcP-3 (n=1)**	88.39 (±2.22)	90.17 (±2.22)	87.00 (±2.74)	86.46 (±2.59)	90.00 (±2.56)	89.99 (±2.43)	82.98 (±3.22)	84.58 (±2.85)	88.33 (±2.87)	80.40 (±3.00)	-			
**PcP-4 (n=3)**	87.01 (±2.16)	88.51 (±2.15)	88.24 (±2.25)	87.32 (±2.28)	90.92 (±2.20)	89.39 (±2.11)	86.62 (±2.56)	87.73 (±2.34)	86.52 (±2.74)	82.99 (±2.63)	84.86 (±2.86)	-		
**PcP-5 (n=1)**	89.61 (±2.17)	90.67 (±2.31)	88.67 (±2.62)	89.80 (±2.23)	93.75 (±2.09)	90.68 (±2.46)	84.52 (±3.01)	88.75 (±2.37)	89.17 (±2.76)	81.42 (±2.97)	85.83 (±3.18)	87.37 (±2.69)	-	
**PcP-7 a (n=5)**	83.66 (±2.67)	84.18 (±2.81)	85.05 (±2.81)	85.63 (±2.49)	87.66 (±2.68)	86.48 (±2.69)	83.66 (±3.08)	85.05 (±2.60)	85.05 (±2.97)	90.68 (±1.61)	80.87 (±3.32)	86.20 (±2.55)	83.31 (±3.05)	-
**PcP-7 b (n=5)**	88.63 (±1.77)	89.34 (±1.91)	88.04 (±2.20)	88.59 (±1.84)	92.40 (±1.70)	91.25 (±1.77)	84.27 (±2.83)	87.70 (±2.10)	89.91 (±2.08)	86.26 (±2.08)	86.11 (±2.60)	87.50 (±2.19)	89.08 (±2.29)	88.51 (±2.11)

[a]*Physalaemus cuvieri* sequences: JF281121.1, JF281117.1, JF281124.1 and JF281119.1, [b]*Physalaemus centralis* sequences: KM361684.1 - KM361688.1, [c]*Physalaemus albonotatus* sequences: KM361689.1 - KM361693.1, [d]*Physalaemus albifrons* sequences: KM361694.1 - KM361698.1, [e]*Physalaemus ephippifer* sequences: KM361699.1 and KM361700.1, [f]*Physalaemus marmoratus* sequences: KM361701.1 - KM361706.1, [g]*Leptodactylus latrans* sequences: KM361718.1 - KM361724.1, [h]*Crossodactylus gaudichaudii* sequences: KM361725.1 and KM361726.1.
