# Supplementary material for: Unraveling the Sex Chromosome Heteromorphism of the Paradoxical Frog Pseudis tocantins
Source: PLoS One. 2016 May 23;11(5):e0156176. doi: 10.1371/journal.pone.0156176 (PMC4877019; doi:10.1371/journal.pone.0156176)
Supplement: S2 Table — (PDF) [file pone.0156176.s002.pdf]

S2 Table. Genetic similarity (%) between the presumed transcribed region of 5S rDNA from several anurans and the more conserved region of the PcP190 sequences of *Physalaemus*, *Leptodactylus*, *Crossodactylus* and *Pseudis*. Numbers in parentheses indicate the standard error. Only complete monomers sequences from PcP190 were used in this analysis. Because no complete monomer of PcP-6 was achieved, this kind of sequence was not included.

PcP190 sequences / 5S rDNA sequences	*Physalaemus* spp.[a]	*Leptodactylus latrans*[b]	*Crossodactylus gaudichaudii*[c]	PcP-1	PcP-2	PcP-3	PcP-4	PcP-5	PcP-7a	PcP-7b
Lithobates pippiens[d]	70.08 (±3.81)	68.16 (±4.15)	67.80 (±3.99)	68.91 (±4.20)	61.45 (±4.00)	68.33 (±4.14)	68.26 (±4.03)	69.75 (±4.11)	65.74 (±4.20)	67.07 (±3.95)
Lithobates catesbeianus[e]	70.13 (±3.84)	68.16 (±4.18)	67.80 (±4.05)	68.91 (±4.22)	61.66 (±4.03)	68.33 (±4.17)	67.95 (±4.14)	69.75 (±4.17)	65.91 (±4.25)	67.07 (±3.98)
Pelophylax lessonae[f]	68.42 (±3.94)	65.45 (±4.33)	67.52 (±4.04)	66.67 (±4.36)	59,87 (±4.17)	69.49 (±4.07)	66.13 (±4.17)	66.67 (±4.30)	62.26 (±4.30)	64.86 (±3.99)
Pelophylax ridibundus[g]	68.42 (±3.94)	65.45 (±4.33)	67.52 (±4.04)	66.67 (±4.36)	59,87 (±4.17)	69.49 (±4.07)	66.13 (±4.17)	66.67 (±4.30)	62.26 (±4.33)	64.86 (±3.99)
Anaxyrus americanus[h]	70.03 (±3.84)	67.43 (±4.21)	67.80 (±4.02)	68.91 (±4.23)	62.29 (±4.01)	69.17 (±4.12)	68.28 (±4.08)	68.91 (±4.18)	65.74 (±4.33)	66.73 (±3.91)
Gastrotheca riobambae[i]	70.27 (±3.81)	67.43 (±4.21)	67.80 (±4.00)	70.59 (±4.16)	64.04 (±3.96)	70.83 (±4.03)	68.28 (±4.02)	68.91 (±4.14)	67.48 (±4.31)	68.36 (±3.85)
Pseudis tocantins 5S-I[j]	61.10 (±5.28)	56.29 (±5.75)	58.67 (±5.30)	59.87 (±5.52)	56.56 (±5.53)	58.55 (±5.52)	59.65 (±5.47)	62.50 (±5.55)	62.76 (±4.27)	60.13 (±4.07)
Pseudis tocantins 5S-II[k]	68.03 (±4.81)	64.76 (±5.14)	65.67 (±4.83)	64.47 (±5.34)	64.39 (±4.97)	72.37 (±4.94)	66.97 (±4.92)	67.11 (±5.10)	66.55 (±4.25)	65.91 (±3.93)
Engystomops petersi 5S-I[l]	67.29 (±3.86)	64.89 (±4.21)	65.25 (±4.04)	68.07 (±4.26)	62.98 (±4.01)	67.50 (±4.17)	65.57 (±4.08)	66.39 (±4.19)	65.97 (±4.19)	66.54 (±3.92)
Engystomops freibergi 5S-I[m]	67.72 (±3.84)	65.85 (±4.15)	65.73 (±4.04)	67.98 (±4.24)	63.48 (±3.97)	67.41 (±4.14)	65.76 (±4.05)	66.29 (±4.16)	66.46 (±4.20)	66.57 (±3.96)
Physalaemus cuvieri 5S-I[n]	68.52 (±4.83)	65.19 (±5.20)	65.33 (±4.88)	70.07 (±5.21)	63.92 (±5.05)	71.38 (±4.99)	65.73 (±4.94)	70.07 (±5.13)	67.30 (±4.17)	66.41 (±4.00)
Engystomops petersi 5S-II[o]	65.24 (±3.93)	64.33 (±4.23)	63.78 (±4.10)	62.82 (±4.31)	61.66 (±4.08)	64.25 (±4.33)	64.82 (±4.17)	62.82 (±4.28)	63.41 (±4.14)	63.74 (±4.05)
Engystomops freibergi 5S-II[p]	66.59 (±3.90)	65.89 (±4.20)	65.07 (±4.07)	64.29 (±4.31)	61.76 (±4.09)	65.42 (±4.29)	65.46 (±4.16)	64.29 (±4.26)	63.57 (±4.20)	64.42 (±3.98)
Physalaemus cuvieri 5S-II[q]	69.06 (±4.62)	66.00 (±5.13)	66.00 (±4.88)	66.45 (±5.26)	58.99 (±5.05)	70.39 (±4.84)	66.00 (±4.89)	69.08 (±4.94)	62.57 (±4.28)	65.03 (±3.89)
Xenopus laevis som[r]	70.15 (±3.84)	68.16 (±4.21)	68.64 (±4.00)	68.91 (±4.21)	62.50 (±4.01)	68.33 (±4.17)	68.28(±4.09)	69.75(±4.13)	66.61 (±4.32)	67.31 (±3.93)
Xenopus laevis ooc[s]	69.58 (±3.77)	68.19 (±4.09)	68.67 (±3.92)	68.58 (±4.08)	62.40 (±3.91)	67.17 (±4.05)	68.31 (±4.00)	69.56 (±4.01)	66.18 (±4.23)	66.71 (±3.87)
Xenopus tropicalis som[t]	69.40 (±3.86)	68.04 (±4.17)	67.37 (±4.05)	69.75 (±4.23)	63.17 (±4.03)	69.17 (±4.16)	67.68 (±4.07)	68.91 (±4.17)	67.48 (±4.27)	68.00 (±4.02)
Xenopus tropicalis ooc[u]	69.40 (±3.81)	66.59 (±4.23)	66.95 (±4.01)	69.75 (±4.15)	61.90 (±4.02)	69.17 (±4.08)	67.37 (±4.06)	68.91 (±4.13)	65.74 (±4.15)	67.77 (±3.94)
Xenopus borealis ooc[v]	69.28 (±3.71)	68.16 (±4.06)	67.80 (±3.90)	68.07 (±4.09)	61.21 (±3.96)	67.50 (±4.03)	67.37 (±4.01)	68.91 (±4.01)	64.00 (±4.21)	65.38 (±4.03)

[a]*Physalaemus* spp. PcP190 sequences: JF281121.1, JF281117.1, JF281124.1, JF281119.1, KM361675.1 - KM361706.1; [b]*Leptodactylus latrans* PcP190 sequences: KM361718.1 - KM361724.1; [c]*Crossodactylus gaudichaudii* PcP190 sequences: KM361725.1 and KM361726.1; [d]*Lithobates pippiens* 5S rRNA gene: X58368.1; [e]*Lithobates catesbeianus* 5S rRNA gene: X58367.1; [f]*Pelophylax lessonae* 5S rRNA gene: FJ572051.1; [g]*Pelophylax ridibundus*

5S rRNA gene: FJ572052.1; [h]*Anaxytus americanus* 5S rRNA gene: X58365.1 (as *Bufo americanus*); [i]*Gastrotheca riobambae* 5S rRNA gene: M74438.1; [j]*Pseudis tocantins* type I 5S rRNA gene: present work; [k]*Pseudis tocantins* type II 5S rRNA gene: present work; [l]*Engystomops petersi* type I 5S rRNA gene: JF325859.1, JF325866.1 and JF325867.1; [m]*Engystomops freibergi* type I 5S rRNA gene: JF325868.1 - JF325870.1; [n]*Physalaemus cuvieri* type I 5S rRNA gene: JF281127.2, JF281128.2, JF281130.2 and JF281131.2; [o]*Engystomops petersi* type II 5S rRNA gene: JF325847.1, JF325848.1 and JF325858.1; [p]*Engystomops freibergi* type II 5S rRNA gene: JF325844.1 and JF325845.1; [q]*Physalaemus cuvieri* type II 5S rRNA gene: JF281132.2 and JF281134.2; [r]*Xenopus laevis* somatic 5S rRNA gene: M35055.1; [s]*Xenopus laevis* oocyte-specific 5S rRNA gene: J01012.1, J01898.1, K02695.1, M10027.1, M10676.1 and M63899.1; [t]*Xenopus tropicalis* somatic 5S rRNA gene: NR 033271.1; [u]*Xenopus tropicalis* oocyte-specific 5S rRNA gene: X12624.1; [v]*Xenopus borealis* 5S rRNA gene: V01425.1 and K01537.1.
